# Benefits of Better Cardiovascular Health for Calcific Aortic Valve Stenosis Stratified by Polygenic Risk Score

**DOI:** 10.1093/gpbjnl/qzaf099

**Published:** 2025-11-06

**Authors:** Yuexin Zhu, Qiuli Chen, Bokang Qiao, Lixin Jia, Haichu Wen, Wei Pan, Yifan Wang, Shuangli Mi, Minxian Wang, Jie Du

**Affiliations:** Beijing Collaborative Innovation Center for Cardiovascular Disorders, MOE Key Laboratory of Remodeling-related Cardiovascular Disease, Beijing Anzhen Hospital, Capital Medical University, Beijing 100029, China; Beijing Institute of Heart, Lung and Blood Vessel Diseases, Beijing 100029, China; Beijing Anzhen Hospital, Capital Medical University, Beijing 100029, China; China National Center for Bioinformation, Beijing 100101, China; Beijing Institute of Genomics, Chinese Academy of Sciences, Beijing 100101, China; University of Chinese Academy of Sciences, Beijing 100049, China; Beijing Collaborative Innovation Center for Cardiovascular Disorders, MOE Key Laboratory of Remodeling-related Cardiovascular Disease, Beijing Anzhen Hospital, Capital Medical University, Beijing 100029, China; Beijing Institute of Heart, Lung and Blood Vessel Diseases, Beijing 100029, China; Beijing Anzhen Hospital, Capital Medical University, Beijing 100029, China; Beijing Collaborative Innovation Center for Cardiovascular Disorders, MOE Key Laboratory of Remodeling-related Cardiovascular Disease, Beijing Anzhen Hospital, Capital Medical University, Beijing 100029, China; Beijing Institute of Heart, Lung and Blood Vessel Diseases, Beijing 100029, China; Beijing Anzhen Hospital, Capital Medical University, Beijing 100029, China; Beijing Collaborative Innovation Center for Cardiovascular Disorders, MOE Key Laboratory of Remodeling-related Cardiovascular Disease, Beijing Anzhen Hospital, Capital Medical University, Beijing 100029, China; Beijing Institute of Heart, Lung and Blood Vessel Diseases, Beijing 100029, China; Beijing Anzhen Hospital, Capital Medical University, Beijing 100029, China; Beijing Collaborative Innovation Center for Cardiovascular Disorders, MOE Key Laboratory of Remodeling-related Cardiovascular Disease, Beijing Anzhen Hospital, Capital Medical University, Beijing 100029, China; Beijing Institute of Heart, Lung and Blood Vessel Diseases, Beijing 100029, China; Beijing Anzhen Hospital, Capital Medical University, Beijing 100029, China; Beijing Collaborative Innovation Center for Cardiovascular Disorders, MOE Key Laboratory of Remodeling-related Cardiovascular Disease, Beijing Anzhen Hospital, Capital Medical University, Beijing 100029, China; Beijing Institute of Heart, Lung and Blood Vessel Diseases, Beijing 100029, China; Beijing Anzhen Hospital, Capital Medical University, Beijing 100029, China; China National Center for Bioinformation, Beijing 100101, China; Beijing Institute of Genomics, Chinese Academy of Sciences, Beijing 100101, China; University of Chinese Academy of Sciences, Beijing 100049, China; China National Center for Bioinformation, Beijing 100101, China; Beijing Institute of Genomics, Chinese Academy of Sciences, Beijing 100101, China; University of Chinese Academy of Sciences, Beijing 100049, China; Beijing Collaborative Innovation Center for Cardiovascular Disorders, MOE Key Laboratory of Remodeling-related Cardiovascular Disease, Beijing Anzhen Hospital, Capital Medical University, Beijing 100029, China; Beijing Institute of Heart, Lung and Blood Vessel Diseases, Beijing 100029, China; Beijing Anzhen Hospital, Capital Medical University, Beijing 100029, China

**Keywords:** Aortic valve stenosis, Polygenic risk score, Primary prevention, Healthy lifestyle, Cardiovascular health

## Abstract

With global aging, the prevalence of calcific aortic valve stenosis (CAVS) has significantly increased, and even mild CAVS elevates mortality risk, highlighting an urgent need for preventive strategies. In this study, we analyzed 153,312 participants from the UK Biobank. We found that a higher Life’s Essential 8 (LE8) score was independently associated with a decreased CAVS risk [hazard ratio (HR)/standard deviation (SD): 0.72, 95% confidence interval (CI): 0.68–0.76, *P* < 2E−16], whereas a higher genetic risk score was independently associated with an increased CAVS risk (HR/SD: 1.63, 95% CI: 1.54–1.71, *P* < 2E−16). Restricted cubic spline analysis revealed approximately linear inverse associations between LE8 score and CAVS risk across all genetic risk groups. Although no multiplicative interaction was found between cardiovascular health (CVH) and genetic risk for CAVS, a significant additive interaction was identified (relative excess risk due to interaction: 3.77, 95% CI: 1.30–8.50). Among participants with high genetic risk, those with ideal CVH had a lower 10-year cumulative CAVS incidence rate than those with poor CVH (0.33% *vs*. 1.80%, *P* < 0.001). Besides, a significant multiplicative interaction was observed between LE8 score and age (*P* = 0.007). Similar trends were also observed for early-onset and late-onset CAVS. In conclusion, regardless of genetic risk groups, a higher LE8 score was associated with a lower CAVS risk in an approximately linear pattern. In particular, high genetic risk and poor CVH had a synergistic effect on CAVS risk, meaning that participants with high genetic risk and poor CVH would amplify their CAVS risk. Therefore, early and sustained optimization of CVH, particularly among those with high genetic risk, is essential to mitigate the risk of CAVS and its subtypes.

## Graphical abstract

**Figure:**
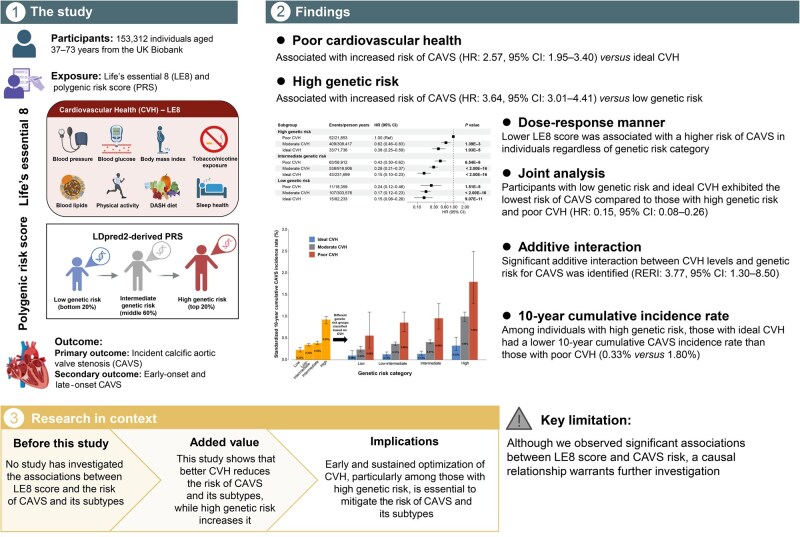

## Introduction

Calcific aortic valve stenosis (CAVS) is characterized by progressive aortic valve calcification, affecting more than 5% of all individuals over the age of 65 years [1]. The global prevalence of CAVS increased by 443% from 1990 to 2019 [2]. Males and females with mild aortic valve stenosis, respectively, have 5.8% and 9.9% higher 5-year mortality rates than those with normal aortic valves [3]. Currently, there are no recommended preventive methods for effectively delaying CAVS onset [4]. Surgical aortic valve replacement (SAVR) and transcatheter aortic valve replacement (TAVR) are the only effective treatments. Given that patient age is a critical factor in the selection of SAVR or TAVR, the American Heart Association (AHA) guideline has recommended that patients should be classified into three categories (< 65, 65–80, and > 80 years of age) [5]. For young patients (< 65 years of age), determining the optimal timing of intervention and selecting the appropriate valve prosthesis are challenging and require comprehensive evaluation [6]. A previous study found that 74.5% of young patients received bioprosthetic valves. Although bioprosthetic valves didn’t require long-term anticoagulation therapy, their average lifetime was shorter than that of mechanical valves, suggesting that young patients might need reintervention in the future [7]. Therefore, preventing the onset of CAVS in young people may be an effective measure to reduce future reintervention risks, simplify treatment decisions, and improve long-term survival.

Genome-wide association studies (GWASs) have identified dozens of risk loci associated with CAVS [8,9]. Polygenic risk score (PRS) can be used as an indicator to identify individuals with higher genetic susceptibility to the disease by combining multiple genetic risk loci [10]. PRSs can help identify individuals with increased genetic predisposition to disease development, yet even highly heritable phenotypes may still be modified by environmental factors [11]. Some preventive measures, such as physical activity and other lifestyle interventions, may reduce the incidence of aortic valve stenosis [12]. However, previous studies have primarily focused on the independent associations between a few lifestyle factors and CAVS risk. Two key gaps remain: first, interactions between multiple lifestyle factors are poorly understood; second, how these factors modify CAVS risk in different genetic risk categories requires further investigation [13,14].

The AHA has recently updated a measure for estimating overall cardiovascular health (CVH) by integrating multiple modifiable risk factors, named Life’s Essential 8 (LE8). The metrics of LE8 include eight important factors: healthy diet, physical activity, tobacco/nicotine exposure, sleep health, body mass index (BMI), blood lipids, blood glucose, and blood pressure [15]. Studies have found that a higher LE8 score — healthier lifestyles — is associated with a lower incidence of various cardiovascular diseases, including coronary heart disease and stroke [16]. However, no study has investigated the associations between the LE8 score and the risk of CAVS and its subtypes (early- and late-onset). Therefore, our hypothesis posits that adopting better CVH levels advocated by LE8 may decrease the risk of CAVS and its subtypes, especially for individuals with high genetic risk.

We conducted a prospective cohort study to investigate (1) the associations between genetic risk, CVH levels, and CAVS risk, (2) the interaction between genetic risk and CVH levels, and (3) whether maintaining ideal CVH can effectively mitigate the elevated CAVS risk observed in individuals with high genetic risk.

## Results

### Baseline characteristics of participants

A total of 153,312 participants retrieved from the UK Biobank (Project ID: 101596) were included in this study (Figure S1) after quality control and exclusion of individuals with missing LE8 variables. The baseline characteristics of the included, excluded, and total populations are summarized in Table S1. The median follow-up duration for the study was 13.27 years, during which 1271 participants were diagnosed with incident CAVS. The mean age of the study population was 55.97 years [standard deviation (SD): 7.96 years], and 82,074 participants (53.53%) were female. Furthermore, 7741 (5.05%), 116,668 (76.10%), and 28,903 (18.85%) participants were categorized as poor, moderate, and ideal CVH, respectively. Compared to participants with poor or moderate CVH, those with ideal CVH were younger, more likely to be female, and had a lower Townsend deprivation index (TDI), higher educational attainment, and higher annual household income (**Table 1**).

**Table 1:** Population characteristics by CVH levels based on LE8 scores

	**Poor CVH** **(LE8 score: 0–49)**	**Moderate CVH** **(LE8 score: 50–79)**	**Ideal CVH** **(LE8 score: 80–100)**
Number	7741	116,668	28,903
Follow-up duration (year): median (IQR)	13.15 (12.56, 13.96)	13.26 (12.65, 14.04)	13.35 (12.74, 14.13)
Age (year): mean ± SD	56.25 ± 7.40	56.65 ± 7.82	53.13 ± 8.05
Female: number (%)	3227 (41.69)	58,597 (50.23)	20,250 (70.06)
Ethnicity: number (%)			
White	7284 (94.59)	111,988 (96.27)	27,845 (96.52)
Others	417 (5.41)	4335 (3.73)	1004 (3.48)
TDI: mean ± SD	−0.79 ± 3.20	−1.67 ± 2.82	−1.71 ± 2.79
Alcohol consumption status: number (%)			
Never	270 (3.49)	3351 (2.87)	972 (3.36)
Previous	367 (4.74)	3315 (2.84)	795 (2.75)
Current	7103 (91.77)	109,958 (94.28)	27,124 (93.88)
Educational attainment: number (%)			
Non-college	5429 (70.52)	67,484 (58.06)	12,526 (43.40)
College	2269 (29.48)	48,748 (41.94)	16,333 (56.60)
Annual household income (£): number (%)			
< 18,000	1642 (23.38)	15,994 (15.11)	2783 (10.50)
18,000–30,999	1722 (24.52)	26,071 (24.62)	5486 (20.70)
31,000–51,999	1877 (26.72)	30,488 (28.80)	7659 (28.90)
52,000–100,000	1448 (20.62)	25,847 (24.41)	7749 (29.23)
> 100,000	335 (4.77)	7479 (7.06)	2829 (10.67)
Aortic valve stenosis: number (%)	126 (1.63)	1054 (0.90)	91 (0.31)
AHA LE8 score: mean ± SD			
Total CVH score	43.95 ± 5.13	67.37 ± 7.54	85.05 ± 4.27
DASH score	16.16 ± 22.25	32.79 ± 28.61	58.01 ± 28.86
Physical activity score	37.49 ± 45.58	87.58 ± 29.69	97.98 ± 9.65
Tobacco/nicotine exposure score	49.78 ± 39.20	77.18 ± 29.35	91.51 ± 16.87
Sleep health score	77.51 ± 24.76	90.31 ± 17.17	95.97 ± 10.77
BMI score	37.82 ± 27.04	69.42 ± 26.70	92.98 ± 13.66
Blood lipid score	31.33 ± 25.77	44.17 ± 26.84	70.50 ± 26.84
Blood glucose score	72.18 ± 28.95	91.85 ± 18.44	98.67 ± 7.60
Blood pressure score	29.32 ± 21.98	45.67 ± 26.22	74.81 ± 25.25
Genetic risk category: number (%)			
High (top 20%)	1711 (22.10)	23,577 (20.21)	5375 (18.60)
Intermediate (middle 60%)	4595 (59.36)	70,023 (60.02)	17,368 (60.09)
Low (bottom 20%)	1435 (18.54)	23,068 (19.77)	6160 (21.31)

*Note*: Continuous variables are presented as mean (SD) or median (IQR). Categorical variables are presented as number (%). CVH levels were categorized as ideal (80**–**100 points), moderate (50–79 points), or poor (0–49 points) according to LE8 criteria. Genetic risk categories were defined using an LDpred2-derived PRS. CVH, cardiovascular health; LE8, life’s essential 8; AHA, American Heart Association; DASH, dietary approaches to stop hypertension; BMI, body mass index; TDI, Townsend deprivation index; IQR, interquartile range; PRS, polygenic risk score; SD, standard deviation.

### Associations of genetic risk and CVH levels with the risk of CAVS and its subtypes

To assess genetic predisposition, four PRSs were constructed using three approaches: clumping and thresholding [C+T; two PRSs based on 29 and 304 single-nucleotide polymorphisms (SNPs), respectively] [17], LDpred2 [18], and Lassosum2 [19]. All four resulting PRSs were all significantly associated with CAVS risk (Table S2) and were approximately normally distributed (Figure S2A–D). When the PRS was analyzed as a standardized continuous variable, the LDpred2-derived PRS showed the highest hazard ratio (HR) for CAVS among the four PRSs (HR/SD: 1.64, 95% confidence interval (CI): 1.55–1.73, *P* < 2E−16) (Table S2). When we conducted further risk stratification, participants were classified into low (bottom 20%), intermediate (middle 60%), and high (top 20%) genetic risk groups based on PRS quintiles. All four PRSs demonstrated similar increasing trends in CAVS risk across higher genetic risk categories (Table S2). Compared to participants with low genetic risk (bottom 20%), those with high genetic risk (top 20%) showed increased CAVS risk across all four PRS construction methods, with HRs of 2.07 (95% CI: 1.73–2.48, *P* = 1.62E−15; Model 1) for the PRS including 29 SNPs, 2.49 (95% CI: 2.07–3.00, *P* < 2E−16; Model 1) for the PRS including 304 SNPs, 3.18 (95% CI: 2.64–3.82, *P* < 2E−16; Model 1) for the PRS constructed by Lassosum2, and 3.68 (95% CI: 3.04–4.45, *P* < 2E−16; Model 1) for the PRS constructed by LDpred2 (Table S2). Among the four PRSs, the LDpred2-derived PRS showed the highest HR for CAVS. Furthermore, when stratifying participants by PRS percentiles, the LDpred2-derived PRS also demonstrated the most distinct gradient in CAVS prevalence, consistent with its better risk discrimination (Figure S2E–H). Therefore, we used the LDpred2-derived PRS for subsequent analysis.

A stepwise increase in CAVS events with increasing genetic risk categories was presented in the cumulative incidence curve (Figure S3A). Compared to participants with low genetic risk (bottom 20%), the HR of those with high genetic risk (top 20%) was 3.64 (95% CI: 3.01–4.41, *P* < 2E−16; Model 2) after further adjustment for CVH levels (**Table 2**, Table S3).

**Table 2:** Associations of genetic risk and CVH levels with CAVS risk

Subgroup	Events/person years	Model 1 HR (95% CI)	*P*	Model 2 HR (95% CI)	*P*
Genetic risk					
Low (bottom 20%)	133/404,168	1.00 (Ref)		1.00 (Ref)	
Intermediate (middle 60%)	644/1,209,517	1.61 (1.33–1.94)	**6.41E–7**	1.60 (1.33–1.93)	**7.94E–7**
High (top 20%)	494/402,006	3.68 (3.04–4.45)	**< 2.00E–16**	3.64 (3.01–4.41)	**< 2.00E–16**
*P* for trend			**< 2.00E–16**		**< 2.00E–16**
CVH level					
Ideal CVH (LE8 score: 80–100)	91/385,669	1.00 (Ref)		1.00 (Ref)	
Moderate CVH (LE8 score: 50–79)	1054/1,530,899	1.69 (1.36–2.10)	**2.10E–6**	1.64 (1.32–2.04)	**6.73E–6**
Poor CVH (LE8 score: 0–49)	126/99,123	2.65 (2.01–3.50)	**6.24E–12**	2.57 (1.95–3.40)	**2.71E–11**
*P* for trend			**2.62E–12**		**1.12E–11**
LE8 score (0–100)	1271/2,015,691	0.97 (0.97–0.98)	**< 2.00E–16**	0.97 (0.97–0.98)	**< 2.00E–16**

*Note*: Genetic risk categories were defined using an LDpred2-derived PRS. CVH levels were categorized by LE8 scores. *P* for trend was calculated by treating the categories as continuous variables, using the median value of each group’s score range. Associations of genetic risk categories and CVH levels with CAVS were evaluated using Cox proportional hazards models. In Model 1, genetic risk was adjusted for age at recruitment, sex, ethnicity, assessment center, TDI, average annual household income, educational attainment, CKD, number of treatments/medications taken, alcohol consumption status, and the first 20 principal components of ancestry; CVH levels were adjusted for the aforementioned covariates expect for assessment center and the first 20 principal components of ancestry. In Model 2, genetic risk was further adjusted for CVH levels; CVH levels were additionally adjusted for genetic risk categories, assessment center, and the first 20 principal components of ancestry. *P* values in bold indicate that the predictor’s effect on the hazard (risk of event) is statistically significant, essentially testing if its HR is different from 1 (which indicates no effect). CI, confidence Interval; HR, hazard ratio; Ref, reference; CAVS, calcified aortic valve stenosis; CKD, chronic kidney disease.

The cumulative incidence curve of CAVS showed incremental increases from ideal to moderate to poor CVH (Figure S3B). The risk of CAVS increased linearly in a dose-response manner across poorer CVH levels (*P* for trend = 2.62E−12) (Table 2). Compared to participants with ideal CVH (LE8 score ≥ 80 points), those with poor CVH (LE8 score < 50 points) had a higher risk of CAVS (HR: 2.65, 95% CI: 2.01–3.50, *P* = 6.24E−12; Model 1). Each 1-point increase in the LE8 score was associated with a 3% reduction in the risk of CAVS (HR: 0.97, 95% CI: 0.97–0.98, *P* < 2.00E−16) (Table 2). These associations remained significant after further adjustment for genetic risk (Table 2, Table S3).

We further conducted secondary analyses to examine potential differences by CAVS disease subtype, specifically early-onset (diagnosis age < 65 years) and late-onset (diagnosis age ≥ 65 years). The early-onset CAVS analysis was restricted to participants younger than 65 years at enrollment (*n* = 129,066), with follow-up ending at the earliest occurrence of CAVS diagnosis, reaching age 65, loss to follow-up, death, or study end. The late-onset CAVS analysis included all participants (*n* = 153,312), and those diagnosed before age 65 were reclassified as controls. In the genetic risk analysis, the association between genetic risk and disease was consistently significant in both early- and late-onset CAVS (Tables S4 and S5). For example, participants with high genetic risk (top 20%) had significantly higher risks of both early-onset (HR: 3.61, 95% CI: 2.28–5.72, *P* = 4.55E−8) (Table S4) and late-onset CAVS (HR: 3.64, 95% CI: 2.95–4.50, *P* < 2.00E−16) (Table S5) compared to those with low genetic risk (bottom 20%) after adjusting for CVH levels. Moreover, participants with poor CVH (LE8 score: 0–49) were associated with higher risks of early-onset (HR: 2.80, 95% CI: 1.52–5.14, *P* = 9.16E−4) (Table S4) and late-onset CAVS (HR: 2.54, 95% CI: 1.86–3.48, *P* = 5.66E−9) (Table S5) after adjusting genetic risk.

### Blood pressure, BMI, tobacco/nicotine exposure, and blood glucose were independently associated with CAVS risk

The associations between each individual LE8 metric and CAVS risk are summarized in **Table 3**. Of the eight metrics in LE8, we observed that four metrics — blood pressure, BMI, tobacco/nicotine exposure, and blood glucose — retained significant independent associations with CAVS risk (low score *vs*. high score, *P* < 0.00625 for each factor) after adjusting for the other seven metrics and genetic risk score. Blood pressure (HR: 1.42, 95% CI: 1.13–1.79, *P* = 0.003), BMI (HR: 1.69, 95% CI: 1.44–1.99, *P* = 1.28E−10), tobacco/nicotine exposure (HR: 1.45, 95% CI: 1.18–1.77, *P* = 3.50E−4), and blood glucose (HR: 1.47, 95% CI: 1.22–1.76, *P* = 4.13E−5) had a higher risk of CAVS in participants with low scores (0–49) compared to those with high scores (80–100) on the corresponding metric. Additionally, genetic correlation analysis revealed significant genetic correlations between CAVS and systolic blood pressure, BMI, and smoking (*P* < 0.05/5 = 0.01 for multiple correction) (Table S6).

**Table 3:** Associations of LE8 metrics with CAVS risk

Lifestyle factor	Events/person years	HR (95% CI)	*P*	PAF (95% CI)
LE8 score				**36.45% (23.87%–49.04%)**
Blood pressure score				**20.36% (3.89%–36.82%)**
High (≥ 80)	84/333,798	1.00 (Ref)		
Intermediate (50–79)	424/978,955	1.10 (0.87–1.39)	0.444	
Low (≤ 49)	763/702,939	**1.42 (1.13–1.79)**	**0.003**	
*P* for trend			**8.35E–5**	
BMI score				**15.68% (6.89%–24.46%)**
High (≥ 80)	291/769,450	1.00 (Ref)		
Intermediate (50–79)	529/843,414	1.09 (0.94–1.27)	0.245	
Low (≤ 49)	451/402,827	**1.69 (1.44–1.99)**	**1.28E–10**	
*P* for trend			**1.35E–13**	
Tobacco/nicotine exposure score				**9.88% (4.00%–15.75%)**
High (≥ 80)	542/1,147,152	1.00 (Ref)		
Intermediate (50–79)	610/690,115	**1.20 (1.06–1.35)**	**0.003**	
Low (≤ 49)	119/178,424	**1.45 (1.18–1.77)**	**3.50E–4**	
*P* for trend			**1.06E–4**	
Blood glucose score				**7.77% (4.07%–11.47%)**
High (≥ 80)	845/1,689,949	1.00 (Ref)		
Intermediate (50–79)	249/232,408	**1.27 (1.10–1.47)**	**0.001**	
Low (≤ 49)	177/93,334	**1.47 (1.22–1.76)**	**4.13E–5**	
*P* for trend			**2.70E–7**	
Blood lipid score				8.34% (−0.77%**–**17.45%)
High (≥ 80)	329/421,685	1.00 (Ref)		
Intermediate (50–79)	155/472,823	1.12 (1.02**–**1.23)	0.097	
Low (≤ 49)	787/1,121,183	**1.19 (1.03–1.36)**	**0.018**	
*P* for trend			**0.016**	
DASH score				4.31% (−6.71%**–**15.33%)
High (≥ 80)	232/410,911	1.00 (Ref)		
Intermediate (50–79)	367/547,655	1.08 (0.91**–**1.27)	0.377	
Low (≤ 49)	672/1,057,126	1.06 (0.91**–**1.24)	0.423	
*P* for trend			0.585	
Physical activity score				−0.40% (−2.78%**–**1.99%)
High (≥ 80)	1,071/1,722,737	1.00 (Ref)		
Intermediate (50–79)	53/80,318	1.02 (0.77**–**1.34)	0.910	
Low (≤ 49)	147/212,636	0.98 (0.82**–**1.17)	0.821	
*P* for trend			0.868	
Sleep health score				0.54% (−2.56%**–**3.64%)
High (≥ 80)	960/1,544,271	1.00 (Ref)		
Intermediate (50–79)	244/368,472	1.08 (0.94**–**1.24)	0.302	
Low (≤ 49)	67/102,948	0.88 (0.68**–**1.12)	0.296	
*P* for trend			0.879	

*Note*: The association of each LE8 metric with CAVS risk was evaluated using Cox proportional hazards models, with all eight LE8 metrics simultaneously included in the multivariable-adjusted models. The association with *P* < 0.00625 (0.05/8 lifestyle factors) was considered significant after Bonferroni correction. The model was adjusted for genetic risk categories, age at recruitment, sex, ethnicity, TDI, average annual household income, educational attainment, CKD, number of treatments/medications taken, alcohol consumption status, assessment center, the first 20 principal components of ancestry, and other lifestyle factors. High, intermediate, and low levels correspond to LE8 component scores of ≥ 80, 50**–**79, and ≤ 49 points, respectively. PAF represents the proportion of CAVS cases that are theoretically preventable if all participants achieve high score (80**–**100 points) for each metric. *P* for trend was calculated by treating the categories as continuous variables, using the median value of each group’s score rang. Bolded values indicate results that are statistically significant. PAF, population attributable fraction.

**Table 4:** Joint associations of genetic risk and CVH levels with CAVS risk

Subgroup	Events/person years	HR (95% CI)	*P*
High genetic risk			
Poor CVH	52/21,853	1.00 (Ref)	
Moderate CVH	409/308,417	0.62 (0.46–0.83)	**1.39E−3**
Ideal CVH	33/71,736	0.38 (0.25–0.59)	**1.93E−5**
Intermediate genetic risk			
Poor CVH	63/58,912	0.43 (0.30–0.62)	**6.54E−6**
Moderate CVH	538/918,906	0.28 (0.21–0.37)	**< 2.00E−16**
Ideal CVH	43/231,699	0.15 (0.10–0.23)	**< 2.00E−16**
Low genetic risk			
Poor CVH	11/18,359	0.24 (0.12–0.46)	**1.51E−5**
Moderate CVH	107/303,576	0.17 (0.12–0.23)	**< 2.00E−16**
Ideal CVH	15/82,233	0.15 (0.08–0.26)	**9.07E−11**

*Note*: Genetic risk categories were defined using an LDpred2-derived PRS. CVH levels were categorized by LE8 scores: poor (0–49), moderate (50–79), and ideal (80**–**100). Joint associations of genetic risk categories and CVH levels with CAVS risk were evaluated using Cox proportional hazards model, which was adjusted for age at recruitment, sex, ethnicity, TDI, average annual household income, educational attainment, CKD, number of treatments/medications taken, alcohol consumption status, assessment center, and the first 20 principal components of ancestry. *P* values in bold indicate that the predictor’s effect on the hazard (risk of event) is statistically significant, essentially testing if its HR is different from 1 (which indicates no effect).

Assuming a causal relationship, the estimated population attributable fraction (PAF) of CAVS due to nonadherence to ideal CVH was 36.45% (95% CI: 23.87%–49.04%). Moreover, for each metric of LE8, maintaining high level (≥ 80 points) for blood pressure, BMI, tobacco/nicotine exposure, and blood glucose could potentially prevent 20.36% (95% CI: 3.89%–36.82%), 15.68% (95% CI: 6.89%–24.46%), 9.88% (95% CI: 4.00%–15.75%), and 7.77% (95% CI: 4.07%–11.47%) of CAVS cases, respectively (Table 3).

### Joint associations of genetic risk and CVH levels with the risk of CAVS and its subtypes

**Figure 1:**
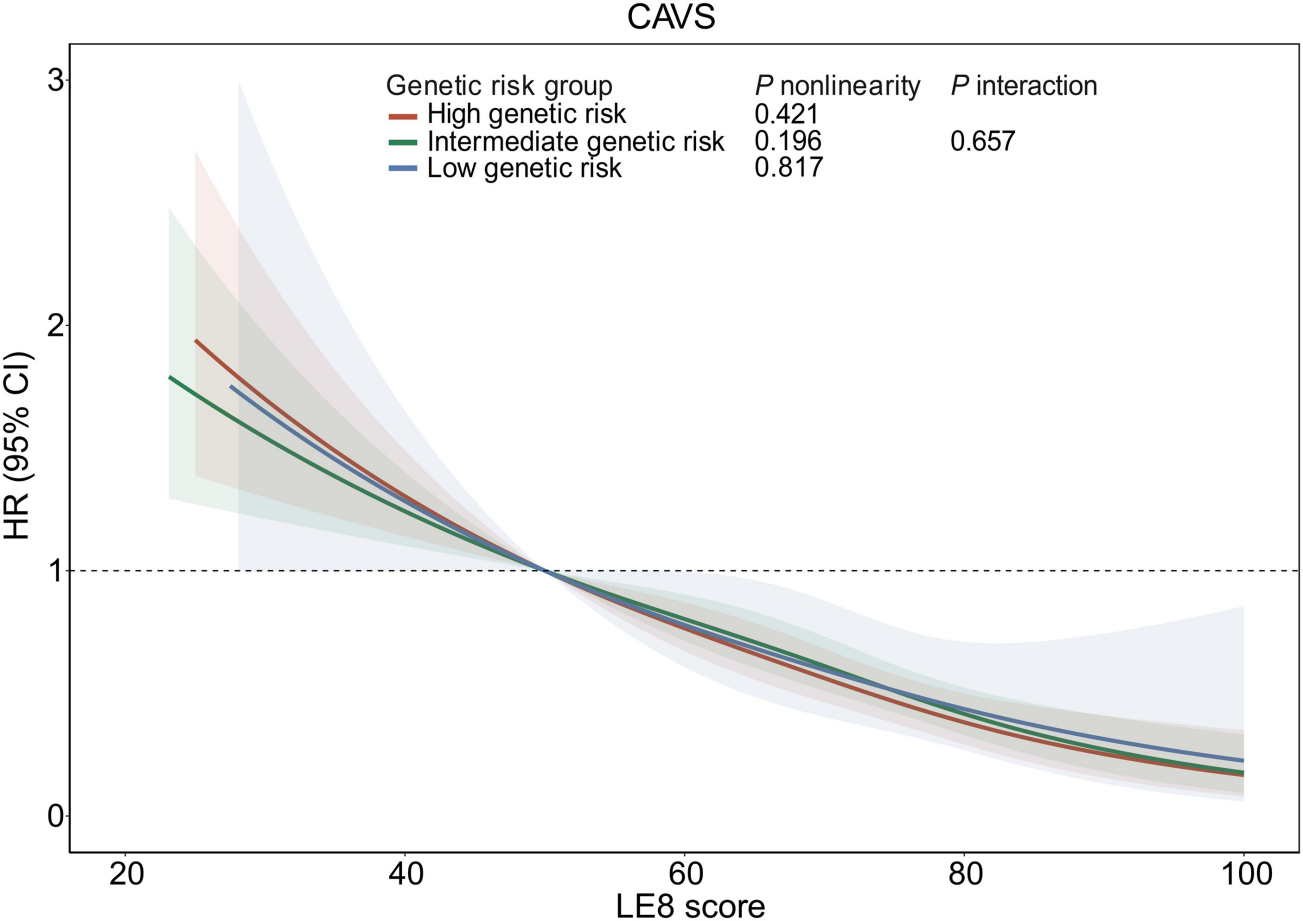
Dose-response association of LE8 score with CAVS risk stratified by genetic risk HRs were calculated using Cox proportional hazards regression model, with adjustment for age at recruitment, sex, ethnicity, TDI, average annual household income, educational attainment, number of treatments/medications taken, CKD, alcohol consumption status, assessment center, and the first 20 principal components of ancestry. Solid lines represent HRs, with shaded areas indicating 95% CI from the restricted cubic spline model. *P* interaction represents the *P* value of multiplicative interaction. *P* nonlinearity represents a statistical *P* value that tests whether the relationship between a continuous variable (*e.g.*, exposure) and an outcome is nonlinear (curved) rather than linear (straight). LE8, life’s essential 8; CAVS, calcified aortic valve stenosis; HR, hazard ratio; TDI, Townsend deprivation index; CKD, chronic kidney disease; CI, confidence interval.

We assessed the joint associations of genetic risk and CVH levels with the risk of CAVS and its subtypes. The approximately linear downward-sloping restricted cubic spline curve illustrated an inverse association between LE8 score and CAVS risk, stratified by genetic risk (**Figure 1**). Although no significant multiplicative interaction was detected (*P* = 0.657) (Figure 1), significant additive interactions were observed between CVH levels and genetic risk for CAVS [relative excess risk due to interaction (RERI): 3.77, 95% CI: 1.30–8.50], early-onset CAVS (RERI: 2.53, 95% CI: 0.26–6.52), and late-onset CAVS (RERI: 4.71, 95% CI: 0.86–12.77) (Tables S7 and S8). Participants with low genetic risk (bottom 20%) and ideal CVH exhibited the lowest risk of CAVS compared to those with high genetic risk (top 20%) and poor CVH (HR: 0.15, 95% CI: 0.08–0.26, *P* = 9.07E−11) (**Table 4**).

**Figure 2:**
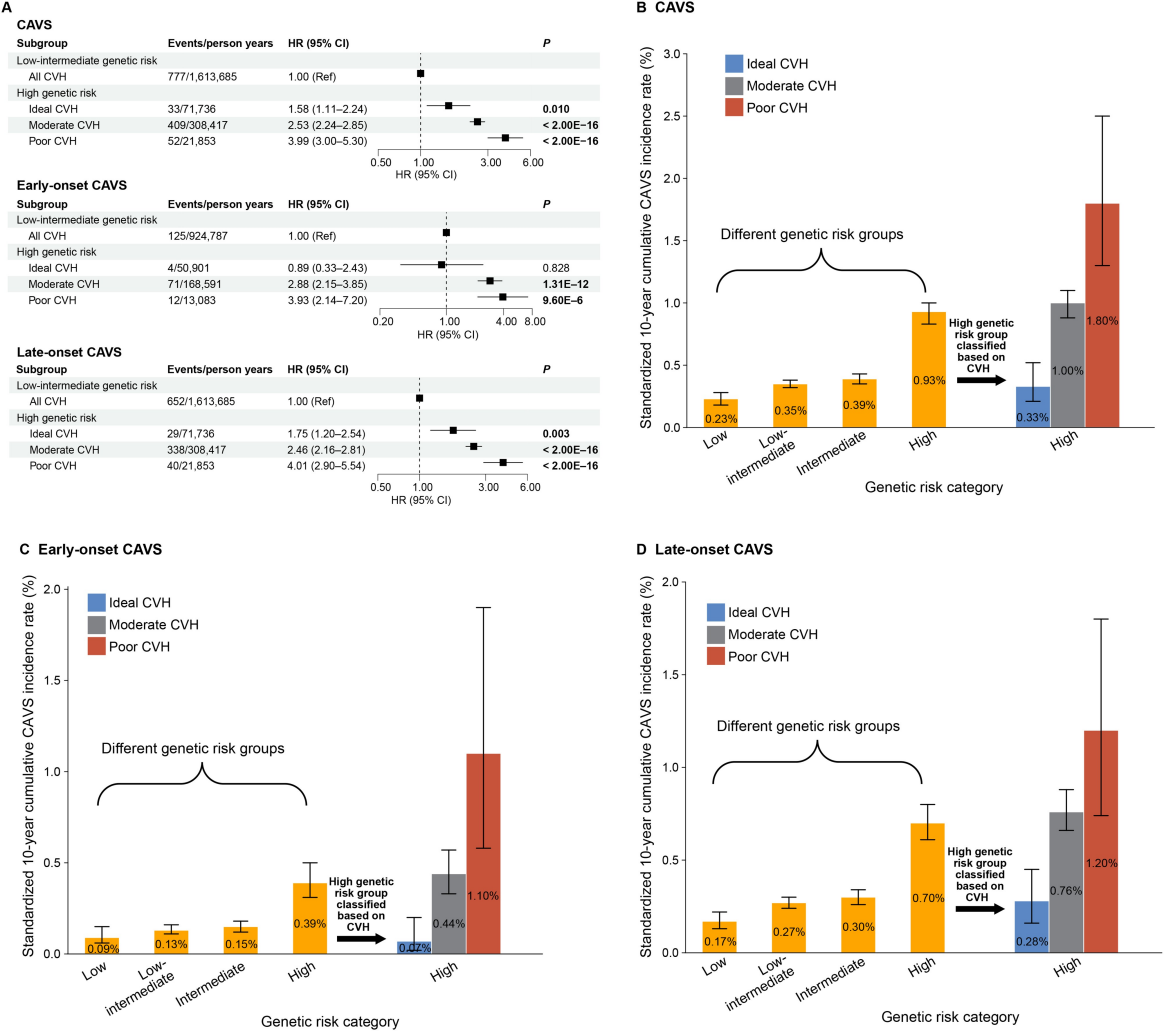
Ten-year cumulative incidence rate of CAVS by genetic risk and CVH levels: high genetic risk ***vs.*** low-intermediate risk **A**. The risk of CAVS and its subtypes (early-onset and late-onset) among different CVH levels in individuals with high genetic risk compared with those with low-intermediate genetic risk. **B**. Bar plot showing the 10-year cumulative incidence rates of CAVS among genetic risk groups (low, low-intermediate, intermediate, high) and within the high genetic risk group stratified by CVH levels. **C**. Bar plot showing the 10-year cumulative incidence rates of early-onset CAVS among genetic risk groups (low, low-intermediate, intermediate, high) and within the high genetic risk group by stratified CVH levels. **D**. Bar plot showing the 10-year cumulative incidence rates of late-onset CAVS among genetic risk groups (low, low-intermediate, intermediate, high) and within the high genetic risk group by stratified CVH levels. CVH, cardiovascular health.

Although higher LE8 score was associated with progressively lower CAVS risk in the high genetic risk group (top 20%) in an approximately linear manner (Figure 1), individuals in this group who maintained ideal CVH still showed significantly higher CAVS risk compared to those with low genetic risk (HR: 2.30, 95% CI: 1.57–3.38, *P* = 2.09E−5; bottom 20%) (Figure S4A) or those with low-intermediate genetic risk (HR: 1.58, 95% CI: 1.11–2.24, *P* = 0.010; bottom 80%) (**Figure 2**A). Notably, while the 10-year cumulative CAVS incidence rate in the high genetic risk group was 0.93%, it varied substantially within this group stratified by CVH levels — individuals with ideal CVH had an incidence rate of 0.33% *vs*. 1.80% in those with poor CVH, corresponding to a 5.45-fold difference (1.80/0.33) (Figure 2B, Figure S5B). These results validated the significant additive interaction between genetic risk and CVH levels, confirming that poorer CVH would further amplify CAVS risk in individuals with high genetic risk.

Next, we performed the secondary analyses for early- and late-onset CAVS. The approximately linear downward-sloping restricted cubic spline curves illustrated inverse associations between LE8 score and the risk of early- and late-onset CAVS across different genetic risk categories (Figure S5C and E). Participants with low genetic risk and ideal CVH also showed the lowest late-onset CAVS risk compared with participants with high genetic risk and poor CVH (Table S9). However, for early-onset CAVS, the lowest risk was observed in participants with low genetic risk and moderate CVH compared with those with high genetic risk and poor CVH (Table S10). Participants with high genetic risk but maintaining ideal CVH still exhibited higher late-onset CAVS risk compared to those with low genetic risk (bottom 20%) (Figure S4C) or combined low-intermediate genetic risk (bottom 80%) (Figure 2A). However, no significant associations were observed for early-onset CAVS between individuals with high genetic risk and ideal CVH and those with low genetic risk (bottom 20%) (Figure S4B) or combined low-intermediate genetic risk (bottom 80%) (Figure 2A). Notably, the 10-year cumulative incidence rate for early-onset CAVS varied significantly in high genetic risk group stratified by CVH levels (0.07% for ideal CVH *vs*. 1.10% for poor CVH) (Figure 2C, Figure S5D). Similarly, for late-onset CAVS, the 10-year cumulative incidence rate for individuals with high genetic risk and ideal CVH was 0.28%, compared to 1.20% for those with high genetic risk and poor CVH (Figure 2D, Figure S5F).

To test the robustness of the associations, we performed a series of sensitivity analyses, including excluding participants diagnosed with CAVS within the first 2 years of follow-up (Table S11), excluding participants with incomplete covariates (Table S12), or adjusting for additional medication use (Table S13). The observed associations remained statistically significant. Furthermore, we examined the cumulative incidence rate of CAVS at multiple time points (3, 5, and 10 years). The 5- and 10-year cumulative incidence rates yielded consistent trends, reinforcing the temporal stability of the associations (Figure S6; Table S14).

### Stratified analyses using potential effect modifiers

Despite variations in baseline risks and effect sizes among different subgroups, a negative association between LE8 score and CAVS risk existed across all of the groups (Figure S7A). Significant multiplicative interactions were observed between LE8 score and age (*P* for multiplicate interaction = 0.007) (Figure S7A) and between LE8 score and sex (*P* for multiplicate interaction = 0.008) (Figure S7A), indicating that the negative association between LE8 score and CAVS risk was stronger in younger individuals and in females. Therefore, we further examined the sex-specific associations between each LE8 metric and CAVS risk and additionally tested for multiplicative interactions between each metric and CAVS risk within each sex group. Four LE8 metrics, including blood pressure, BMI, tobacco/nicotine exposure, and blood glucose, were still independently associated with CAVS risk in both males and females after adjusting for genetic risk and the other seven LE8 metrics (Table S15). Although no significant multiplicative interactions were detected between these four LE8 metrics and CAVS risk within either sex group, the strength of associations seemed to be stronger in females than in males. Notably, blood lipids were significantly associated with CAVS in females (HR: 0.82; 95% CI: 0.74–0.91; *P* = 2.78E−4) (Table S15) but not in males (HR: 0.91; 95% CI: 0.84–0.98; *P* = 0.011) (Table S15), suggesting a potential female-specific effect.

Besides, we further explored whether genetic risk, which was identified by LDpred2-derived PRS based on GWAS summary data from Million Veteran Program (MVP), differed by sex. First, we separately performed sex-stratified subgroup analyses to assess the associations between genetic risk and CAVS risk in males and females, and we observed that genetic risk was significantly associated with CAVS risk in males or females (Table S16). Besides, no significant multiplicative interaction was observed between sex and genetic risk score (*P* for interaction = 0.656) (Table S16). We further evaluated the associations between genetic risk and the risk of early-onset or late-onset CAVS. We observed similar associations between genetic risk and the risk of CAVS subtypes (Table S17).

Additionally, there were no significant interactions between CVH levels and TDI or educational attainment in relation to CAVS risk (all *P* for multiplicate interaction > 0.05) (Figure S7A).

## Discussion

In this large prospective cohort study, we found that higher PRS_CAVS_ and lower LE8 score were independently associated with increased risk of CAVS and its subtypes. Compared to participants with ideal CVH, the CAVS risk of those with poor CVH was increased by 165%. Besides, the dose-response analysis further revealed negative associations between LE8 score and the risk of CAVS and its subtypes across all genetic risk categories. We observed significant additive interactions between genetic risk and CVH levels, suggesting that the increase in CAVS risk (as reflected by HRs) from ideal to poor CVH was greater among individuals with high genetic risk than those with lower genetic risk. Additionally, we observed significant multiplicative interactions between CVH and sex or age. Poorer CVH in females conferred greater CAVS risk than in males, and in younger individuals than in older individuals. Among participants with high genetic risk, maintaining ideal CVH can change the 10-year cumulative incidence rate of CAVS from 0.93% to 0.33%.

Although no previous studies have analyzed the impact of LE8 score on the risk of CAVS and its subtypes, the EPIC-Norfolk cohort study, which used Life’s Simple 7 (LS7) to assess CVH, found that participants in the top quartile of LS7 score had a lower risk of CAVS compared to those in the bottom quartile [20]. However, the quartile grouping of LS7 may be data-dependent because it is derived using population-specific cut-points, which makes it challenging to compare the results with other research and apply them in population education [21]. Besides, our results revealed that the association between LE8 score and CAVS was stronger in female and younger participants. Although four LE8 metrics — blood pressure, BMI, tobacco/nicotine exposure, and blood glucose — were significantly associated with CAVS risk in both males and females, the point estimates appeared to be consistently smaller in females than in males. Additionally, blood lipid levels were significantly associated with CAVS risk only in females, which may further explain the stronger impact of poorer CVH on CAVS risk in females. Additionally, the National Health and Nutrition Examination Survey (NHANES) revealed that the overall CVH of individuals tends to decline with age in the general population [22]. Despite previous studies showing that males were more likely to have CAVS than females, the prevalence of CAVS in females has been observed to increase significantly with age, and most patients over 80 years old with aortic valve stenosis were female [23]. Therefore, maintaining ideal CVH during early life is particularly important for preventing CAVS in females. Both sexes are encouraged to adopt the ideal CVH in early life to optimally reduce the prevalence of CAVS.

Although our analyses suggested that improvements in CVH were associated with reduced CAVS risk in other ethnic groups, caution is warranted when interpreting these findings. The “other” ethnicity category in our study comprised multiple distinct subpopulations, including Black, Asian, and mixed ethnic backgrounds. However, due to the limited representation of these groups in the UK Biobank and the low number of CAVS events observed within each subgroup (Table S18), our study lacked sufficient power to generate robust, stratified conclusions for non-White populations. Therefore, the generalizability of our findings to other ethnic groups remains uncertain, and future studies are needed to validate these associations in more diverse and adequately powered multi-ethnic cohorts.

Our joint analysis of genetic risk and CVH levels demonstrated a significant additive interaction, whereby individuals with high genetic risk and poor CVH experienced greater CAVS risk. Besides, to investigate the impact of CVH levels on early-onset CAVS, we classified CAVS into early-onset (< 65 years) and late-onset (≥ 65 years) CAVS based on the diagnosis age. We observed that adopting better CVH levels could significantly reduce the risk of early-onset CAVS. Besides, we further analyzed the relationship between each metric of LE8 and CAVS to identify the key modifiable risk factors in LE8 that could reduce the risk of CAVS. We found that four modifiable risk factors (blood pressure, BMI, tobacco/nicotine exposure, and blood glucose) were associated with CAVS risk. Additionally, prior evidence has supported potential causal relationships between some of these metrics and aortic valve stenosis. For example, elevated BMI and blood pressure have been identified as likely causal risk factors through Mendelian randomization and large-scale cohort studies [24,25]. Although current evidence does not suggest a clear causal relationship between smoking and aortic valve stenosis [26], an association between diabetes and aortic valve disease has been reported [27]. Furthermore, although the genetic susceptibility of an individual is determined, disease risk also heavily depends on various modifiable factors, such as environmental exposure and medical history [28]. In our study, we observed significant genetic correlations between CAVS and three modifiable LE8 metrics, including systolic blood pressure, BMI, and smoking, indicating partial shared genetic architecture.

Several significant risk loci associated with CAVS, such as those in *LPA* and *FTO*, are related to lipid metabolism and obesity, respectively [9], which could be offset by maintaining ideal CVH. Currently, several mechanisms could explain the aforementioned associations. The key pathogenesis of CAVS is endothelial dysfunction caused by disturbed blood flow, lipid accumulation, chronic inflammation, and valve interstitial cells undergoing osteogenic reprogramming [29]. Hypertension may accelerate the progression of CAVS by increasing mechanical stress on the valve leaflets [30], and obesity could significantly affect rising blood pressure and upregulate adipose-derived autotaxin, which could induce local inflammation and calcification in the aortic valve [31]. Hyperglycemia increases the formation and amount of advanced glycation end products, which promotes oxidative stress [27]. Tobacco, rich in toxic oxidizing chemicals and free radicals, could alter lipoprotein composition and induce a proinflammatory state in the valves [32]. These findings show that the independent impacts and interactions with the other risk factors contributed to the incidence of CAVS. Although no significant association was observed between a healthy diet [33], blood lipid [34], adequate physical activity [35], and healthy sleep [14] and the risk of CAVS, which was consistent with the results of most previous studies, these risk factors should still be strictly managed, because every metric of LE8 interacts with each other. Besides, the estimated PAF of CAVS due to nonadherence to ideal CVH in our study was stronger than each LE8 metric. Therefore, implementing a more aggressive overall CVH management strategy could maximize the prevention of CAVS in participants with high genetic risk. In addition, based on the identification of more potential risk variants within a larger sample cohort, the PRS could be more effective in identifying high genetic risk carriers [36]. Compared with a previous study, which evaluated the association between lifestyle, genetic risk, and aortic valve stenosis [13], we constructed the PRS based on a larger sample size cohort (MVP) to more accurately evaluate the genetic susceptibility of individuals and better assess the preventive benefits of adhering to ideal CVH in populations with high genetic risk.

However, several limitations of our study should be acknowledged. First, the observational nature of the study precludes causal inference. Although we observed significant associations between LE8 metrics and CAVS risk, a causal relationship warrants further investigation. Second, the generalizability of our findings may be limited, as the majority of participants were of European ancestry. Therefore, the results may not be applicable to non-European populations, and further studies in more ethnically diverse cohorts are needed to confirm the associations across different ancestral backgrounds. Third, selection bias may have occurred, as participants in the UK Biobank tend to be healthier and more health-conscious than the general population. This “healthy volunteer” bias may limit the external validity of our findings. Fourth, despite the inclusion of additional risk variants from large-scale GWAS, some genetic susceptibilities, such as structural variants, may have remained undetected, potentially limiting the comprehensiveness of the PRS. Finally, several metrics of the LE8 score, particularly physical activity and dietary intake, were self-reported and may be subject to recall bias. Moreover, these lifestyle factors could have changed over the follow-up period. However, any misclassification is likely nondifferential due to the prospective design, which would tend to attenuate the observed associations toward the null.

## Materials and methods

### Study participants

The study design and population of the UK Biobank have been outlined previously [37]. A total of 502,366 participants were recruited from the UK Biobank (Project ID: 101596; https://www.ukbiobank.ac.uk/). We sequentially excluded participants who withdrew from the study (*n* = 178), those with missing or low-quality genotyping data (*n* = 16,231), those with a medical history of rheumatic fever, rheumatic heart disease, congenital valve malformations, or aortic valve stenosis (*n* = 1710) [38], and those with missing data related to LE8 (*n* = 330,935). Finally, a total of 153,312 participants were included in this study (Figure S1).

### Genetic risk profiling

We constructed PRSs for CAVS using summary statistics from the multi-ancestry GWAS conducted by the MVP, which is predominantly composed of individuals of European ancestry (72.41%) [39]. First, we generated conventional PRSs through C+T at two stringency levels: (1) genome-wide significant SNPs (*P* ≤ 5 × 10^−8^, 29 variants; Table S19) and (2) suggestive SNPs (*P* ≤ 1 × 10^−4^, 304 variants; Table S20), both LD-pruned (*r*^2^ < 0.01) and weighted by MVP effect sizes [9]. Second, We processed HapMap3 SNPs through LDpred2 [18] and Lassosum2 [19]. For LDpred2, default parameters were employed as per a previous study [40]: the proportion of assumed causal variants (cut-offs at *P* = 1.0 × 10^−4^, 1.8 × 10^−4^, 3.2 × 10^−4^, 5.6 × 10^−4^, 1.0 × 10^−3^, 1.8 × 10^−3^, 3.2 × 10^−3^, 5.6 × 10^−3^, 1.0 × 10^−2^, 1.8 × 10^−2^, 3.2 × 10^−2^, 5.6 × 10^−2^, 1.0 × 10^−1^, 1.8 × 10^−1^, 3.2 × 10^−1^, 5.6 × 10^−1^, and 1), the scale of heritability (s = 0.7, 1, and 1.4), and the option of whether to apply a sparse LD matrix. We applied Lassosum2 with s values of 0.001, 0.01, 0.1, and 1, along with 30 λ values. All PRSs were validated in an independent UK Biobank subset (*n* = 332,173). The PRS with the best stratification ability was used in the downstream analysis, and PRSs were classified by centiles to examine changes in the prevalence of CAVS as the PRS centiles increased.

### Genome-wide association studies

A total of 407,330 quality-controlled individuals from the UK Biobank were initially selected for GWAS (Figure S8). The association of each genetic variant with CAVS was assessed using REGENIE [41], adjusting for age, sex, genotype measurement, and the first 10 genetic principal components. The resulting GWAS summary statistics were subsequently used to estimate genetic correlations through linkage disequilibrium score regression (LDSC) (Figure S9) [42].

### CVH assessment with LE8

CVH was evaluated using the LE8 score, which includes eight metrics. In addition to the healthy diet based on the dietary approaches to stop hypertension (DASH) diet score, the other seven metrics were processed according to the original guidelines. We assessed the dietary intake levels based on the modified DASH diet score (without considering sodium intake) [43]. Medication use was incorporated into the scoring of several metrics. For blood pressure and blood lipids, a 20-point deduction was applied if participants were using related medications. For blood glucose, individuals using glucose-lowering drugs were classified as having diabetes. The detailed definitions and scoring of every LE8 metric are outlined in Table S21.

The LE8 metrics and the overall score were evaluated at baseline, with every metric scored from 0 to 100 points. The overall LE8 score of each individual was derived by summing the scores of the eight metrics and dividing by 8. Based on the AHA’s categorical recommendation, CVH was categorized as poor (0**–**49), moderate (50**–**79), and ideal (80**–**100) based on the overall LE8 score [15].

### Follow-up and outcome assessment

This study focused on CAVS and its subtypes (early-onset and late-onset). Diagnostic information was obtained using a combination of primary care data, linked hospital admission records, and death registry data. Detailed disease phenotypes are provided in Table S22. The follow-up duration for every participant was measured from the time of enrollment to the earliest occurrence of any of the following: CAVS diagnosis, loss to follow-up, death, or the end of the follow-up period (May 31, 2022 for Wales; August 31, 2022 for Scotland; and October 30, 2022 for England). Besides, CAVS was classified into early-onset (diagnosed at age < 65 years) and late-onset (diagnosed at age ≥ 65 years). For early-onset CAVS analysis, we only included the participants who were younger than 65 years at enrollment (*n* = 129,066), and the follow-up duration for each participant was measured from the time of enrollment to the earliest occurrence of CAVS diagnosis, loss to follow-up, death, reaching age 65 during follow-up, or the end of the follow-up period (dates as mentioned above). For late-onset CAVS analysis, participants diagnosed at age < 65 years were regarded as controls.

### Covariates

Covariates were collected through questionnaires or interviews and included: age at recruitment (< 65 years, ≥ 65 years), sex (male, female), ethnicity (White, others), TDI (divided into tertiles), self-reported average annual household income (£; < 18,000, 18,000–30,999, 31,000–51,999, 52,000–100,000, > 100,000), alcohol consumption status (never, past, current), educational attainment (non-college, college), chronic kidney disease (CKD), number of treatments/medications taken, assessment center, and the first 20 principal components of ancestry. CKD was diagnosed based on self-reported questionnaire responses, hospital admission records, and death registry data (Table S22). Missing data for ethnicity (0.29% missing), TDI (0.11% missing), self-reported average annual household income (9.07% missing), educational attainment (0.34% missing), and alcohol consumption status (0.04% missing) were imputed using multiple imputation methods [44].

### Statistical analysis

According to the CVH levels, baseline characteristics were described as mean (SD) or median (interquartile range) for continuous variables and as number (%) for categorical variables. Baseline characteristics between the individuals included and excluded are presented in Table S1.

Cox proportional hazards models were used to evaluate the associations of genetic risk, CVH levels, and combined genetic risk and CVH levels (participants with high genetic risk and poor CVH as references) with CAVS and its subtypes. The models were adjusted for age at recruitment, sex, ethnicity, TDI, educational attainment, average annual household income, alcohol consumption status, CKD, and number of treatments/medications taken. Specifically, Model 1 assessed CVH levels (adjusted for all the covariates listed above) and genetic risk groups (adjusted for all the covariates listed above, as well as assessment center and the first 20 principal components of ancestry), respectively. Model 2 evaluated the joint associations by further adjusting genetic risk groups for CVH levels and adjusting CVH levels for genetic risk groups, assessment center, and first 20 principal components of ancestry, while maintaining all other covariates from Model 1. The proportional hazards assumption was confirmed using the Schoenfeld residuals method, and no observed violations were detected (*P* > 0.05). Subsequently, we performed a multivariable Cox model simultaneously including all eight metrics of LE8 to evaluate their independent associations with incident CAVS, addressing potential correlations among these metrics. Bonferroni correction was applied for multiple test correction. The PAF and its 95% CI were calculated to estimate the proportion of CAVS cases in this study that could theoretically be prevented if all participants adhered to ideal CVH or were in a high-score group of the single LE8 metric [45].

We further calculated the dose-response associations between LE8 score and the risk of CAVS and its subtypes using restricted cubic spline models stratified by genetic risk. Additionally, we evaluated the multiplicative and additive interactions of CVH levels and genetic risk categories on the risk of CAVS and its subtypes. We calculated the RERI and 95% CI using the MOVER method for additive interactions [46].

Additionally, for CAVS and its subtypes, we categorized high genetic risk carriers (top 20%) by CVH levels and compared their risk of CAVS and its subtypes with that of carriers with low (bottom 20%) or low-intermediate genetic risk (remaining 80%) [47]. The 10-year cumulative incidence rates of CAVS and its subtypes were also calculated using the cumulative incidence function of competing risk regression.

We further explored the robustness of the associations between CVH levels and CAVS across subgroups stratified by age at enrollment (< 65 years, ≥ 65 years), sex (male, female), ethnicity (White, others), TDI (divided into tertiles), educational attainment (non-college, college) and additional medication use. All tests were two-sided, with *P* < 0.05 considered statistically significant. All analyses were performed using R software (v4.4.1).

## Ethical statement

The UK Biobank was approved by the North West Research Ethics Committee (Approval No. 21/NW/0157) and conducted according to the principles of the Declaration of Helsinki. All participants gave written informed consent before enrolment.

## Code availability

The R code is available at BioCode at the National Genomics Data Center (NGDC), China National Center for Bioinformation (CNCB) (BioCode: BT007925), which is publicly accessible at https://ngdc.cncb.ac.cn/biocode/tool/BT007925.

## Supplementary Material

qzaf099_Supplementary_Data

## CRediT author statement

**Yuexin Zhu:** Conceptualization, Data curation, Formal analysis, Investigation, Methodology, Resources, Software, Validation, Visualization, Writing – original draft, Writing – review & editing. **Qiuli Chen:** Formal analysis, Investigation, Methodology, Writing – review & editing. **Bokang Qiao:** Software, Writing – review & editing. **Lixin Jia:** Project administration, Supervision, Writing – review & editing. **Haichu Wen:** Resources, Software, Visualization. **Wei Pan:** Visualization, Software. **Yifan Wang:** Software, Visualization. **Shuangli Mi:** Writing – review & editing. **Minxian Wang:** Conceptualization, Methodology, Project administration, Supervision, Writing – review & editing. **Jie Du:** Conceptualization, Funding acquisition, Project administration, Supervision, Writing – review & editing. All authors have read and approved the final manuscript.

## References

[1] Boskovski MT , Gleason TG. Current therapeutic options in aortic stenosis. Circ Res 2021;128:1398–417. 33914604 10.1161/CIRCRESAHA.121.318040

[2] Martin SS , Aday AW, Almarzooq ZI, Anderson CAM, Arora P, Avery CL, et al 2024 Heart Disease and Stroke Statistics: a report of US and global data from the American Heart Association. Circulation 2024;149:e347–913. 38264914 10.1161/CIR.0000000000001209 PMC12146881

[3] Stewart S , Afoakwah C, Chan YK, Strom JB, Playford D, Strange GA. Counting the cost of premature mortality with progressively worse aortic stenosis in Australia: a clinical cohort study. Lancet Healthy Longev 2022;3:e599–606. 36102774 10.1016/S2666-7568(22)00168-4 PMC9484033

[4] Messika-Zeitoun D , Baumgartner H, Burwash IG, Vahanian A, Bax J, Pibarot P, et al Unmet needs in valvular heart disease. Eur Heart J 2023;44:1862–73. 36924203 10.1093/eurheartj/ehad121

[5] Writing Committee Members, Otto CM, Nishimura RA, Bonow RO, Carabello BA, Erwin JP 3rd, et al 2020 ACC/AHA guideline for the management of patients with valvular heart disease: a report of the American College of Cardiology/American Heart Association Joint Committee on Clinical Practice Guidelines. J Am Coll Cardiol 2021;77:e25–197. 10.1016/j.jacc.2020.11.018 33342586

[6] Baman JR , Medhekar AN, Malaisrie SC, McCarthy P, Davidson CJ, Bonow RO. Management challenges in patients younger than 65 years with severe aortic valve disease: a review. JAMA Cardiol 2023;8:281–9. 36542365 10.1001/jamacardio.2022.4770

[7] Mehta CK , Liu TX, Bonnell L, Habib RH, Kaneko T, Flaherty JD, et al Age-stratified surgical aortic valve replacement for aortic stenosis. Ann Thorac Surg 2024;118:430–8. 38286202 10.1016/j.athoracsur.2024.01.013

[8] Small AM , Peloso GM, Linefsky J, Aragam J, Galloway A, Tanukonda V, et al Multiancestry genome-wide association study of aortic stenosis identifies multiple novel loci in the Million Veteran Program. Circulation 2023;147:942–55. 36802703 10.1161/CIRCULATIONAHA.122.061451 PMC10806851

[9] Yu Chen H , Dina C, Small AM, Shaffer CM, Levinson RT, Helgadottir A, et al Dyslipidemia, inflammation, calcification, and adiposity in aortic stenosis: a genome-wide study. Eur Heart J 2023;44:1927–39. 37038246 10.1093/eurheartj/ehad142 PMC10232274

[10] Small AM , Melloni GEM, Kamanu FK, Bergmark BA, Bonaca MP, O’Donoghue ML, et al Novel polygenic risk score and established clinical risk factors for risk estimation of aortic stenosis. JAMA Cardiol 2024;9:357–66. 38416462 10.1001/jamacardio.2024.0011 PMC10902779

[11] Harden KP. Genetic determinism, essentialism and reductionism: semantic clarity for contested science. Nat Rev Genet 2023;24:197–204. 36316396 10.1038/s41576-022-00537-x

[12] Li Z , Cheng S, Guo B, Ding L, Liang Y, Shen Y, et al Wearable device-measured moderate to vigorous physical activity and risk of degenerative aortic valve stenosis. Eur Heart J 2025;46:649–64. 38953786 10.1093/eurheartj/ehae406 PMC11825145

[13] Jia C , Zeng Y, Huang X, Yang H, Qu Y, Hu Y, et al Lifestyle patterns, genetic susceptibility, and risk of valvular heart disease: a prospective cohort study based on the UK Biobank. Eur J Prev Cardiol 2023;30:1665–73. 37259902 10.1093/eurjpc/zwad177

[14] Huang N , Zhuang Z, Liu Z, Huang T. Observational and genetic associations of modifiable risk factors with aortic valve stenosis: a prospective cohort study of 0.5 million participants. Nutrients 2022;14:2273. 35684074 10.3390/nu14112273 PMC9182826

[15] Lloyd-Jones DM , Allen NB, Anderson CAM, Black T, Brewer LC, Foraker RE, et al Life’s Essential 8: updating and enhancing the American Heart Association’s construct of cardiovascular health: a presidential advisory from the American Heart Association. Circulation 2022;146:e18–43. 35766027 10.1161/CIR.0000000000001078 PMC10503546

[16] Wang X , Ma H, Li X, Heianza Y, Manson JE, Franco OH, et al Association of cardiovascular health with life expectancy free of cardiovascular disease, diabetes, cancer, and dementia in UK adults. JAMA Intern Med 2023;183:340–9. 36848126 10.1001/jamainternmed.2023.0015 PMC9972243

[17] International Schizophrenia Consortium , Purcell SM, Wray NR, Stone JL, Visscher PM, O’Donovan MC, et al Common polygenic variation contributes to risk of schizophrenia and bipolar disorder. Nature 2009;460:748–52. 19571811 10.1038/nature08185 PMC3912837

[18] Prive F , Arbel J, Vilhjalmsson BJ. LDpred2: better, faster, stronger. Bioinformatics 2021;36:5424–31. 33326037 10.1093/bioinformatics/btaa1029 PMC8016455

[19] Prive F , Arbel J, Aschard H, Vilhjalmsson BJ. Identifying and correcting for misspecifications in GWAS summary statistics and polygenic scores. HGG Adv 2022;3:100136. 36105883 10.1016/j.xhgg.2022.100136 PMC9465343

[20] Perrot N , Boekholdt SM, Mathieu P, Wareham NJ, Khaw KT, Arsenault BJ. Life’s simple 7 and calcific aortic valve stenosis incidence in apparently healthy men and women. Int J Cardiol 2018;269:226–8. 30054144 10.1016/j.ijcard.2018.07.107 PMC6481556

[21] Bennette C , Vickers A. Against quantiles: categorization of continuous variables in epidemiologic research, and its discontents. BMC Med Res Methodol 2012;12:21. 22375553 10.1186/1471-2288-12-21 PMC3353173

[22] Lloyd-Jones DM , Ning H, Labarthe D, Brewer L, Sharma G, Rosamond W, et al Status of cardiovascular health in US adults and children using the American Heart Association’s new “Life’s Essential 8” metrics: prevalence estimates from the National Health and Nutrition Examination Survey (NHANES), 2013 through 2018. Circulation 2022;146:822–35. 35766033 10.1161/CIRCULATIONAHA.122.060911

[23] Coffey S , Roberts-Thomson R, Brown A, Carapetis J, Chen M, Enriquez-Sarano M, et al Global epidemiology of valvular heart disease. Nat Rev Cardiol 2021;18:853–64. 34172950 10.1038/s41569-021-00570-z

[24] Kaltoft M , Langsted A, Nordestgaard BG. Obesity as a causal risk factor for aortic valve stenosis. J Am Coll Cardiol 2020;75:163–76. 31948645 10.1016/j.jacc.2019.10.050

[25] Rahimi K , Mohseni H, Kiran A, Tran J, Nazarzadeh M, Rahimian F, et al Elevated blood pressure and risk of aortic valve disease: a cohort analysis of 5.4 million UK adults. Eur Heart J 2018;39:3596–603. 30212891 10.1093/eurheartj/ehy486 PMC6186276

[26] Larsson SC , Mason AM, Back M, Klarin D, Damrauer SM, Million Veteran P, et al Genetic predisposition to smoking in relation to 14 cardiovascular diseases. Eur Heart J 2020;41:3304–10. 32300774 10.1093/eurheartj/ehaa193 PMC7544540

[27] Rawshani A , Sattar N, McGuire DK, Wallstrom O, Smith U, Boren J, et al Left-sided degenerative valvular heart disease in type 1 and type 2 diabetes. Circulation 2022;146:398–411. 35678729 10.1161/CIRCULATIONAHA.121.058072

[28] Lewis CM , Vassos E. Polygenic risk scores: from research tools to clinical instruments. Genome Med 2020;12:44. 32423490 10.1186/s13073-020-00742-5 PMC7236300

[29] Small AM , Yutzey KE, Binstadt BA, Voigts Key K, Bouatia-Naji N, Milan D, et al Unraveling the mechanisms of valvular heart disease to identify medical therapy targets: a scientific statement from the American Heart Association. Circulation 2024;150:e109–28. 38881493 10.1161/CIR.0000000000001254 PMC11542557

[30] Moncla LM , Briend M, Bosse Y, Mathieu P. Calcific aortic valve disease: mechanisms, prevention and treatment. Nat Rev Cardiol 2023;20:546–59. 36829083 10.1038/s41569-023-00845-7

[31] Rogers MA , Aikawa E. An (auto)taxing effort to mechanistically link obesity and calcific aortic valve disease. JACC Basic Transl Sci 2020;5:898–900. 33016951 10.1016/j.jacbts.2020.04.016 PMC7524779

[32] Dudzinski DM , O’Gara PT. Association of cigarette smoking with degenerative aortic valve disease. Circ Cardiovasc Imaging 2019;12:e009441. 31405291 10.1161/CIRCIMAGING.119.009441

[33] Janzi S , Dias JA, Martinsson A, Sonestedt E. Association between dietary fiber intake and risk of incident aortic stenosis. Nutr Metab Cardiovasc Dis 2020;30:2180–5. 32907763 10.1016/j.numecd.2020.07.015

[34] Chan KL , Teo K, Dumesnil JG, Ni A, Tam J, Investigators A. Effect of lipid lowering with rosuvastatin on progression of aortic stenosis: results of the aortic stenosis progression observation: measuring effects of rosuvastatin (ASTRONOMER) trial. Circulation 2010;121:306–14. 20048204 10.1161/CIRCULATIONAHA.109.900027

[35] Sarajlic P , Wolk A, Back M, Larsson SC. Physical activity does not reduce aortic valve stenosis incidence. Circ J 2018;82:2372–4. 29998916 10.1253/circj.CJ-18-0598

[36] Uffelmann E , Huang QQ, Munung NS, de Vries J, Okada Y, Martin AR, et al Genome-wide association studies. Nat Rev Methods Primers 2021;1:59.

[37] Sudlow C , Gallacher J, Allen N, Beral V, Burton P, Danesh J, et al UK biobank: an open access resource for identifying the causes of a wide range of complex diseases of middle and old age. PLoS Med 2015;12:e1001779. 25826379 10.1371/journal.pmed.1001779 PMC4380465

[38] Theriault S , Li Z, Abner E, Luan J, Manikpurage HD, Houessou U, et al Integrative genomic analyses identify candidate causal genes for calcific aortic valve stenosis involving tissue-specific regulation. Nat Commun 2024;15:2407. 38494474 10.1038/s41467-024-46639-4 PMC10944835

[39] Verma A , Huffman JE, Rodriguez A, Conery M, Liu M, Ho YL, et al Diversity and scale: genetic architecture of 2068 traits in the VA Million Veteran Program. Science 2024;385:eadj1182. 39024449 10.1126/science.adj1182 PMC12857194

[40] Patel AP , Wang M, Ruan Y, Koyama S, Clarke SL, Yang X, et al A multi-ancestry polygenic risk score improves risk prediction for coronary artery disease. Nat Med 2023;29:1793–803. 37414900 10.1038/s41591-023-02429-x PMC10353935

[41] Mbatchou J , Barnard L, Backman J, Marcketta A, Kosmicki JA, Ziyatdinov A, et al Computationally efficient whole-genome regression for quantitative and binary traits. Nat Genet 2021;53:1097–103. 34017140 10.1038/s41588-021-00870-7

[42] Bulik-Sullivan B , Finucane HK, Anttila V, Gusev A, Day FR, Loh PR, et al An atlas of genetic correlations across human diseases and traits. Nat Genet 2015;47:1236–41. 26414676 10.1038/ng.3406 PMC4797329

[43] Djousse L , Ho YL, Nguyen XT, Gagnon DR, Wilson PWF, Cho K, et al DASH score and subsequent risk of coronary artery disease: the findings from Million Veteran Program. J Am Heart Assoc 2018;7:e008089. 10.1161/JAHA.117.008089 PMC6015298 29680824

[44] Esmaeilzadeh M , Urzua Fresno CM, Somerset E, Shalmon T, Amir E, Fan CS, et al A combined echocardiography approach for the diagnosis of cancer therapy-related cardiac dysfunction in women with early-stage breast cancer. JAMA Cardiol 2022;7:330–40. 35138325 10.1001/jamacardio.2021.5881 PMC8829754

[45] Dahlqwist E , Zetterqvist J, Pawitan Y, Sjolander A. Model-based estimation of the attributable fraction for cross-sectional, case-control and cohort studies using the R package AF. Eur J Epidemiol 2016;31:575–82. 26992709 10.1007/s10654-016-0137-7

[46] Zou GY. On the estimation of additive interaction by use of the four-by-two table and beyond. Am J Epidemiol 2008;168:212–24. 18511428 10.1093/aje/kwn104

[47] Kim MS , Shim I, Fahed AC, Do R, Park WY, Natarajan P, et al Association of genetic risk, lifestyle, and their interaction with obesity and obesity-related morbidities. Cell Metab 2024;36:1494–503.e3. 38959863 10.1016/j.cmet.2024.06.004 PMC12285577

