# Supplementary material for: Benefits of Better Cardiovascular Health for Calcific Aortic Valve Stenosis Stratified by Polygenic Risk Score
Source: Genomics Proteomics Bioinformatics. 2025 Nov 6;23(5):qzaf099. doi: 10.1093/gpbjnl/qzaf099 (PMC12812169; doi:10.1093/gpbjnl/qzaf099)
Supplement: qzaf099_Supplementary_Data [file qzaf099_supplementary_data.zip › Table S21.docx]

**Table S21 Definitions of the LE8’s metrics used in the UK Biobank study**

| CVH metric | Method of measurement | Scoring of CVH metric | |
| --- | --- | --- | --- |
|  |  | Points | Status |
| DASH diet score | Using dietary data collected from 24-h dietary recalls (1-5 times) conducted using the Oxford WebQ between 2009 and 2012. | 100  80  50  25  0 | 95th percentile (ideal diet)  75th–94th percentile  50th–74th percentile  25th–49th percentile  1st–24th percentile (least ideal quartile) |
| Physical activity score | Self-reported minutes of moderate and vigorous physical activity per week. | 100  90  80  60  40  20  0 | ≥ 150 minutes  120-149 minutes  90-119 minutes  60-89 minutes  30-59 minutes  1-29 minutes  0 minutes |
| Tobacco/nicotine exposure score | Self-reported use of cigarettes or secondhand smoke exposure.  Furthermore, for participants without information on the duration since quitting smoking, we consider individuals who indicated they 'smoked occasionally in the past' as equivalent to 'Former smoker, quit 1–<5 years', and those who indicated they 'just tried once or twice in the past' as equivalent to 'Former smoker, quit ≥ 5 years'. | 100  75  50  25  0 | Never smoker  Former smoker, quit ≥ 5 years  Former smoker, quit 1 – 5 years  Former smoker, quit < 1 years  Current smoker |
|  |  | **If the score is not 0, deduct 20 points for living with active indoor smokers at home.** | |
| Sleep health score | Self-reported average hours of sleep per night. | 100  90  70  40  20  0 | ≥7 to <9 h/day  ≥9 to <10 h/day  ≥6 to <7 h/day  ≥5 to <6 or ≥10 h/day  ≥4 to <5 h/day  <4 h/day |
| Body mass index score | Body mass index (kg/m2). | 100  70  30  15  0 | < 25 kg/m2  25.0-29.9 kg/m2  30.0-34.9 kg/m2  35.0-39.9 kg/m2  ≥ 40.0 kg/m2 |
| Blood lipids score | Plasma total and HDL cholesterol to estimate non-HDL cholesterol.  If the score is not 0, deduct 20 points if participants were in treated level. | 100  60  40  20  0 | < 130 mg/dL  130-159 mg/dL  160-189 mg/dL  190-219 mg/dL  ≥ 220 mg/dL |
| Blood glucose score | Casual HbA1c.  **Diabetes was defined using a combination of self-reported questionnaire responses, hospital admission records, and death registry data. Additionally, participants who were prescribed glucose-lowering medications were classified as having diabetes** | 100  60  40  30  20  10  0 | No history of diabetes HbA1c < 5.7 %  No diabetes and HbA1c 5.7–6.4%  Diabetes with HbA1c < 7.0 %  Diabetes with HbA1c 7.0–7.9 %  Diabetes with HbA1c 8.0–8.9 %  Diabetes with HbA1c 9.0–9.9 %  Diabetes with HbA1c ≥ 10.0 % |
| Blood pressure score | Appropriately measured systolic and diastolic blood pressure.  **If the score is not 0, deduct 20 points if participants were in treated level.** | 100  75  50  25  0 | < 120 and < 80 mm Hg  120-129 and < 80 mm Hg  130-139 or 80-89 mm Hg  140-159 or 90-99 mm Hg  ≥ 160 or ≥ 100 mm Hg |
