# Supplementary material for: Benefits of Better Cardiovascular Health for Calcific Aortic Valve Stenosis Stratified by Polygenic Risk Score
Source: Genomics Proteomics Bioinformatics. 2025 Nov 6;23(5):qzaf099. doi: 10.1093/gpbjnl/qzaf099 (PMC12812169; doi:10.1093/gpbjnl/qzaf099)
Supplement: qzaf099_Supplementary_Data [file qzaf099_supplementary_data.zip › Table S22.docx]

**Table S22 Disease definitions used in the UK Biobank study**

| **Diagnosis** | **Self-report (field 20002)** | **ICD 9 (field 41271)** | **ICD 10 (field 41270/40001)** |
| --- | --- | --- | --- |
| Nonrheumatic aortic valve stenosis | 1490 | - | I35.0, I35.2 |
| Congenital malformations/stenosis of aortic valves | - | - | Q23.0, Q23.8, Q23.9 |
| Chronic rheumatic heart disease | - | 393-398 | I05-I09 |
| Rheumatic fever | - | 390-392 | I00-I02 |
| Diabetes mellitus | 1220, 1222, 1223 | 250 | E10-E14 |
| Chronic kidney disease | - | - | N18, N18.1, N18.2, N18.3, N18.4, N18.5, N18.9 |
