# Supplementary material for: Benefits of Better Cardiovascular Health for Calcific Aortic Valve Stenosis Stratified by Polygenic Risk Score
Source: Genomics Proteomics Bioinformatics. 2025 Nov 6;23(5):qzaf099. doi: 10.1093/gpbjnl/qzaf099 (PMC12812169; doi:10.1093/gpbjnl/qzaf099)
Supplement: qzaf099_Supplementary_Data [file qzaf099_supplementary_data.zip › Supplementary material captions.docx]

**Supplementary materials**

**Figure S1 Flowchart of study population selection in the UK Biobank study**

**Figure S2** **Distribution of PRS and associations between different PRSs with CAVS prevalence**

**A.–D.** Distribution of standardized polygenic risk scores (PRSs) for CAVS constructed using 29 SNPs (A), 304 SNPs (B), LDpred2 (C), and lassosum2 (D). **E.–H.** Association between PRS percentiles and CAVS prevalence for 29-SNP PRS (E), 304-SNP PRS (F), lassosum2-derived PRS (G), and LDpred2-derived PRS (H). CAVS, calcific aortic valve stenosis; PRS, polygenic risk score; SNP, single-nucleotide polymorphism; CVH, cardiovascular health; LE8, Life’s Essential 8; HR, hazard ratio; CI, confidence interval; Ref, reference.

**Figure S3 Cumulative incidence curves of CAVS by genetic risk and CVH levels**

**A.** Cumulative incidence curves of CAVS across PRS. **B.** Cumulative incidence curves of CAVS across CVH levels classified as ideal, moderate, and poor based on Life’s Essential 8 (LE8). The shadow of curves represents 95% confidence interval. CAVS, calcific aortic valve stenosis; PRS, polygenic risk score; CVH, cardiovascular health.

**Figure S4 Risks of CAVS, early-onset CAVS and late-onset CAVS by CVH levels among individuals with high genetic risk (low genetic risk group as reference)**

**A.** Risk of CAVS across different CVH levels among individuals with high genetic risk (top 20%), compared to those with low genetic risk (reference group). **B.** Risk of early-onset CAVS across different CVH levels in the high genetic risk group compared to low genetic risk. **C.** Risk of late-onset CAVS across different CVH levels in the high genetic risk group compared to low genetic risk. Model was adjusted for age at recruitment, sex, ethnicity, townsend deprivation index, average annual household income, educational attainment, number of treatments/medications taken, chronic kidney disease, alcohol consumption status, assessment center and first 20 principal components of ancestry. CAVS, calcific aortic valve stenosis; PRS, polygenic risk score; CVH, cardiovascular health; CI, confidence interval; HR, hazard ratio; REF, reference.

**Figure S5 Dose–response associations and 10-year cumulative incidence rate of CAVS and its subtypes by CVH and genetic risk**

**A.** Restricted cubic spline showing the dose–response association between LE8 score and CAVS risk within each genetic risk category. **B.** Ten-year cumulative incidence rate of CAVS across groups defined by genetic risk (low, low-intermediate, intermediate, high) and CVH levels (poor, moderate, ideal) within the high genetic risk group. **C.** Restricted cubic spline showing the dose–response association between LE8 score and early-onset CAVS risk within each genetic risk category. **D.** Ten-year cumulative incidence rate of early-onset CAVS across groups defined by genetic risk (low, low-intermediate, intermediate, high) and CVH levels (poor, moderate, ideal) within the high genetic risk group. **E.** Restricted cubic spline showing the dose–response association between LE8 score and late-onset CAVS risk within each genetic risk category. **F.** Ten-year cumulative incidence rate of late-onset CAVS across groups defined by genetic risk (low, low-intermediate, intermediate, high) and CVH levels (poor, moderate, ideal) within the high genetic risk group. Hazard ratios (HRs) were calculated using Cox proportional hazards models adjusted for age at recruitment, sex, ethnicity, Townsend deprivation index, average annual household income, educational attainment, chronic kidney disease, number of treatments/medications taken, alcohol consumption status, assessment center, and the first 20 principal components of ancestry. Solid lines represent HRs; shaded areas indicate 95% confidence intervals. CAVS, calcific aortic valve stenosis; CVH, cardiovascular health; LE8, Life’s Essential 8; HR, hazard ratio; CI, confidence interval; PRS, polygenic risk score.

**Figure S6 3-, 5-, and 10-year cumulative incidence rate of CAVS by CVH levels and genetic risk**

**A.** Cumulative incidence rate of CAVS at 3, 5, and 10 years in the total population. **B.** 3-year cumulative incidence rate of CAVS across genetic risk categories, with further CVH stratification in the high genetic risk group. **C.** 5-year cumulative incidence rate of CAVS across genetic risk categories, with further CVH stratification in the high genetic risk group. **D.** 10-year cumulative incidence rate of CAVS across genetic risk categories, with further CVH stratification in the high genetic risk group. CAVS, calcific aortic valve stenosis; CVH, cardiovascular health; LE8, Life’s Essential 8; HR, hazard ratio; CI, confidence interval; PRS, polygenic risk score.

**Figure S7 Stratified analyses of the associations between LE8 score and CAVS risk by potential effect modifiers**

**A.** Associations between LE8 score and CAVS risk across subgroups defined by age, sex, ethnicity, TDI, and education. **B.** The risk of CAVS by CVH level among individuals with high genetic risk, stratified by age at recruitment compared with those with low and intermediate genetic risk within the same age group. **C.** The risk of CAVS by CVH level among individuals with high genetic risk, stratified by sex compared with those with low and intermediate genetic risk within the same sex. Hazard ratios (HRs) were calculated using Cox proportional hazards models adjusted for genetic risk, average annual household income, chronic kidney disease, number of treatments/medications taken, alcohol consumption status, assessment center, and the first 20 principal components of ancestry. Age-stratified models were additionally adjusted for sex, ethnicity, Townsend deprivation index, and educational attainment. Sex-stratified models for age at recruitment, ethnicity, Townsend deprivation index, and educational attainment. Ethnicity-stratified models for age at recruitment, sex, Townsend deprivation index, and educational attainment. Education-stratified models for age at recruitment, sex, ethnicity, and Townsend deprivation index. CAVS, calcific aortic valve stenosis; CVH, cardiovascular health; LE8, Life’s Essential 8; HR, hazard ratio; CI, confidence interval; PRS, polygenic risk score.

**Figure S8 Flowchart of study population selection for Genome-Wide Association Studies of CAVS**

**Figure S9 Manhattan plots of CAVS in the overall population**

**Table S1 Baseline characteristics of participants included, excluded, and the total population from the UK Biobank cohort**

**Table S2 Associations of different polygenic risk scores with CAVS**

**Table S3 Associations of genetic risk score and LE8 score with CAVS**

**Table S4 Associations of genetic risk and CVH levels based on the LE8 score with early-onset CAVS (diagnosis age < 65; *n* = 129,066)**

**Table S5** **Associations of genetic risk and CVH levels based on the LE8 score with late-onset CAVS (diagnosis age ≥ 65; *n* = 153,312)**

**Table S6 Genetic Correlations between CAVS and five phenotypic traits**

**Table S7 RERI and 95% CI for additive interaction between CVH levels and genetic risk (low genetic risk and ideal CVH are the references)**

**Table S8 RERI and 95% CI for additive interaction between CVH levels and genetic risk (intermediate genetic risk and ideal CVH are the references)**

**Table S9 Risk of late-onset CAVS (diagnosis age ≥ 65) by joint categorization for genetic risk and CVH levels (*n* =153,312)**

**Table S10 Risk of early-onset CAVS (diagnosis age < 65) by joint categorization for genetic risk and CVH levels (*n =* 129,066)**

**Table S11 Risk of CAVS by joint categorization for genetic risk and CVH levels after excluding incident CAVS within the first 2 years of follow-up (*n* = 153,262)**

**Table S12 Risk of CAVS by joint categorization for genetic risk and CVH levels after excluding participants with incomplete covariates (*n* = 138,681)**

**Table S13 Associations between CVH levels and risk of CAVS after adjustment for additional medication use**

**Table S14** **Distribution of CAVS events at 3-, 5-, and 10-year follow-up by CVH levels and genetic risk**

**Table S15** **Sex-stratified analyses of the associations between each LE8 metric and the risk of CAVS in the overall population after adjusting genetic risk**

**Table S16 Sex-stratified associations between genetic risk (LDpred2-derived PRS based on MVP GWAS summary) with CAVS**

**Table S17 Sex-stratified associations between genetic risk (LDpred2-derived PRS based on MVP GWAS summary) with early-onset and late-onset CAVS**

**Table S18 Distribution of CAVS events across ethnic groups in the included cohort (*n* = 153,312)**

**Table S19 Single-nucleotide polymorphisms used to build the genetic risk score including 29 SNPs (*P* ≤ 5 × 10^‒8^)**

**Table S20 Single-nucleotide polymorphisms used to build the genetic risk score including 304 SNPs (*P* ≤ 1 × 10^‒4^)**

**Table S21 Definitions of the LE8’s metrics used in the UK Biobank study**

**Table S22 Disease definitions used in the UK Biobank study**
