# Supplementary material for: Benefits of Better Cardiovascular Health for Calcific Aortic Valve Stenosis Stratified by Polygenic Risk Score
Source: Genomics Proteomics Bioinformatics. 2025 Nov 6;23(5):qzaf099. doi: 10.1093/gpbjnl/qzaf099 (PMC12812169; doi:10.1093/gpbjnl/qzaf099)
Supplement: qzaf099_Supplementary_Data [file qzaf099_supplementary_data.zip › Table S1.docx]

**Table S1 Baseline characteristics of participants included, excluded, and the total population from the UK Biobank cohort**

|  |  | **Included** | **Excluded** | **Total** |
| --- | --- | --- | --- | --- |
| N |  | 153,312 | 330,935 | 484,247 |
| Age (years), mean ± SD |  | 55.97 ± 7.96 | 56.78 ± 8.14 | 56.52 ± 8.09 |
| Female, No. (%) |  | 82,074 (53.53) | 180,816 (54.64) | 262,890 (54.29) |
| Ethnicity, No. (%) | White | 147,117 (95.96) | 309,208 (93.43) | 456,325 (94.23) |
|  | Others | 5756 (3.75) | 19,886 (6.01) | 25,642 (5.30) |
| BMI (kg/m^2^), mean ± SD |  | 26.84 ± 4.54 | 27.68 ± 4.87 | 27.41 ± 4.78 |
| TDI, mean ± SD |  | −1.63 ± 2.84 | −1.16 ± 3.18 | −1.31 ± 3.09 |
| Alcohol consumption status, No. (%) | Never | 4593 (3.00) | 16,740 (5.06) | 21,333 (4.41) |
|  | Previous | 4477 (2.92) | 12,811 (3.87) | 17,288 (3.57) |
|  | Current | 144,185 (94.05) | 300,222 (90.72) | 444,407 (91.77) |
| Educational attainment, No. (%) | Non-college | 85,439 (55.73) | 235,832 (71.26) | 321,271 (66.34) |
|  | College | 67,350 (43.93) | 89,411 (27.02) | 156,761 (32.37) |
| Annual household income (£), No. (%) | < 18,000 | 20,419 (13.32) | 73,324 (22.16) | 93,743 (19.36) |
|  | 18,000–30,999 | 33,279 (21.71) | 71,819 (21.70) | 105,098 (21.70) |
|  | 31,000–51,999 | 40,024 (26.11) | 67,851 (20.50) | 107,875 (22.28) |
|  | 52,000–100,000 | 35,044 (22.86) | 49,170 (14.86) | 84,214 (17.39) |
|  | > 100,000 | 10,643 (6.94) | 11,785 (3.56) | 22,428 (4.63) |
| AHA life’s essential 8 score, mean ± SD | Total CVH score | 69.52 ± 11.40 | - | 69.52 ± 11.40 |
| Follow-up duration (years), median (IQR) |  | 13.27 (12.66, 14.05) | 13.77 (12.99, 14.36) | 13.63 (12.88, 14.29) |

*Note*: Continuous variables are presented as mean (SD) or median (IQR). Categorical variables are presented as N (%). AHA, American Heart Association; BMI, Body Mass Index; CVH, Cardiovascular Health; TDI, Townsend deprivation index.
