# Supplementary material for: Benefits of Better Cardiovascular Health for Calcific Aortic Valve Stenosis Stratified by Polygenic Risk Score
Source: Genomics Proteomics Bioinformatics. 2025 Nov 6;23(5):qzaf099. doi: 10.1093/gpbjnl/qzaf099 (PMC12812169; doi:10.1093/gpbjnl/qzaf099)
Supplement: qzaf099_Supplementary_Data [file qzaf099_supplementary_data.zip › Table S2.docx]

**Table S2 Associations of different polygenic risk scores with CAVS**

| **Subgroup** | **Events/person years** | **Model 1 HR (95% CI)** | ***P* value** | **Model 2 HR (95% CI)** | ***P* value** |
| --- | --- | --- | --- | --- | --- |
| **LDpred2** |  |  |  |  |  |
| Low (bottom 20%) | 133/404,168 | Ref |  | Ref |  |
| Intermediate (middle 60%) | 644/1,209,517 | 1.61 (1.33, 1.94) | **6.41E–7** | 1.60 (1.33, 1.93) | **7.94E–7** |
| High (top 20%) | 494/402,006 | 3.68 (3.04, 4.45) | **< 2E–16** | 3.64 (3.01, 4.41) | **< 2E–16** |
| P trend |  |  | **< 2E–16** |  | **< 2E–16** |
| Continuous PRS (per 1-SD increase) | 1271/2,015,691 | 1.64 (1.55, 1.73) | **< 2E–16** | 1.63 (1.54, 1.72) | **< 2E–16** |
|  |  |  |  |  |  |
| **Lassosum2** |  |  |  |  |  |
| Low (bottom 20%) | 152/404,027 | Ref |  | Ref |  |
| Intermediate (middle 60%) | 659/1,209,542 | 1.45 (1.22, 1.73) | **3.59E–5** | 1.45 (1.22, 1.73) | **3.73E–5** |
| High (top 20%) | 460/402,123 | 3.18 (2.64, 3.82) | **< 2E–16** | 3.16 (2.63, 3.79) | **< 2E–16** |
| P trend |  |  | **< 2E–16** |  | **< 2E–16** |
| Continuous PRS (per 1-SD increase) | 1271/2,015,691 | 1.60 (1.51, 1.69) | **< 2E–16** | 1.59 (1.50, 1.68) | **< 2E–16** |
|  |  |  |  |  |  |
| **PRS including 29 SNPs** |  |  |  |  |  |
| Low (bottom 20%) | 175/403,097 | Ref |  | Ref |  |
| Intermediate (middle 60%) | 713/1,209,776 | 1.31 (1.11, 1.54) | **0.002** | 1.30 (1.11, 1.54) | **0.002** |
| High (top 20%) | 383/402,818 | 2.07 (1.73, 2.48) | **1.62E–15** | 2.07 (1.73, 2.48) | **2.10E–15** |
| P trend |  |  | **< 2E–16** |  | **< 2E–16** |
| Continuous PRS (per 1-SD increase) | 1271/2,015,691 | 1.29 (1.23, 1.37) | **< 2E–16** | 1.29 (1.22, 1.36) | **< 2E–16** |
|  |  |  |  |  |  |
| **PRS including 304 SNPs** |  |  |  |  |  |
| Low (bottom 20%) | 154/403,485 | Ref |  | Ref |  |
| Intermediate (middle 60%) | 723/1,209,398 | 1.54 (1.30, 1.84) | **9.71E–7** | 1.55 (1.30, 1.84) | **9.57E–7** |
| High (top 20%) | 394/402,808 | 2.49 (2.07, 3.00) | **< 2E–16** | 2.48 (2.05, 2.98) | **< 2E–16** |
| P trend |  |  | **< 2E–16** |  | **< 2E–16** |
| Continuous PRS (per 1-SD increase) | 1271/2,015,691 | 1.39 (1.31, 1.47) | **< 2E–16** | 1.38 (1.31, 1.46) | **< 2E–16** |

*Note*: We used cox proportional hazards models to evaluate the associations between different PRSs and CAVS. Polygenic risk scores were standardized (mean = 0, SD = 1), and hazard ratios were estimated per 1-SD increase. Model 1 was adjusted for age at recruitment, sex, ethnicity, assessment center, townsend deprivation index, average annual household income, educational attainment, chronic kidney disease, number of treatments/medications taken, alcohol consumption status, and the first 20 principal components of ancestry. Model 2 was additionally adjusted for CVH levels based on Model 1. The trend test used the median value of each group instead of the original group. HR, hazard ratio; CI, confidence interval; PRS, polygenic risk score; CAVS, calcific aortic valve stenosis.
