# Supplementary material for: Benefits of Better Cardiovascular Health for Calcific Aortic Valve Stenosis Stratified by Polygenic Risk Score
Source: Genomics Proteomics Bioinformatics. 2025 Nov 6;23(5):qzaf099. doi: 10.1093/gpbjnl/qzaf099 (PMC12812169; doi:10.1093/gpbjnl/qzaf099)
Supplement: qzaf099_Supplementary_Data [file qzaf099_supplementary_data.zip › Table S3.docx]

**Table S3 Associations of genetic risk score and LE8 score with CAVS**

| **Subgroup** | **HR/SD (95% CI)** | **Regression coefficient** | **Standard error of the coefficient** | ***P* value** |
| --- | --- | --- | --- | --- |
| Genetic risk score | 1.63 (1.54, 1.71) | 0.48 | 0.03 | < 2E–16 |
| LE8 score | 0.72 (0.68, 0.76) | –0.33 | 0.03 | < 2E–16 |

*Note*: The genetic risk score was computed by using LDpred2. Genetic risk score and LE8 score were standardized (mean = 0, SD = 1). We used cox proportional hazards models to evaluate the associations between genetic risk score and LE8 score with CAVS. The genetic risk score and LE8 score were included in the same model and mutually adjusted for each other. The model was also adjusted for age at recruitment, sex, ethnicity, assessment center, townsend deprivation index, average annual household income, educational attainment, chronic kidney disease, number of treatments/medications taken, alcohol consumption status, and the first 20 principal components of ancestry.
