# Supplementary material for: Benefits of Better Cardiovascular Health for Calcific Aortic Valve Stenosis Stratified by Polygenic Risk Score
Source: Genomics Proteomics Bioinformatics. 2025 Nov 6;23(5):qzaf099. doi: 10.1093/gpbjnl/qzaf099 (PMC12812169; doi:10.1093/gpbjnl/qzaf099)
Supplement: qzaf099_Supplementary_Data [file qzaf099_supplementary_data.zip › Table S4.docx]

**Table S4 Associations of genetic risk and CVH levels based on the LE8 score with early-onset CAVS (diagnosis age < 65; *n* = 129,066)**

| **Subgroup** | **Events/person years** | **Model 1 HR (95% CI)** | ***P* value** | **Model 2 HR (95% CI)** | ***P* value** |
| --- | --- | --- | --- | --- | --- |
| **Genetic risk** |  |  |  |  |  |
| Low (bottom 20%) | 23/231,036 | Ref |  | Ref |  |
| Intermediate (middle 60%) | 102/693,752 | 1.48 (0.94, 2.33) | 0.090 | 1.47 (0.93, 2.31) | 0.096 |
| High (top 20%) | 87/232,575 | 3.67 (2.31, 5.81) | **3.12E–8** | 3.61 (2.28, 5.72) | **4.55E–8** |
| P trend |  |  | **2.26E–11** |  | **3.49E–11** |
|  |  |  |  |  |  |
| **CVH levels by LE8 scores** |  |  |  |  |  |
| Ideal CVH | 19/268,492 | Ref |  | Ref |  |
| Moderate CVH | 163/831,087 | 1.76 (1.08, 2.85) | **0.022** | 1.71 (1.05, 2.78) | **0.030** |
| Poor CVH | 30/57,783 | 2.91 (1.58, 5.35) | **5.88E–4** | 2.80 (1.52, 5.14) | **9.16E–4** |
| P trend |  |  | **4.41E–4** |  | **6.91E–4** |
| LE8 score (0-100 points) | 212/1,157,362 | 0.97 (0.96, 0.98) | **4.29E–7** | 0.97 (0.96, 0.98) | **1.35E–6** |

*Note*: Genetic risk categories were defined using an LDpred2-derived PRS. CVH levels were categorized as ideal (80–100 points), moderate (50–79 points), or poor (0–49 points) according to LE8 criteria. The metrics of LE8 include healthy diet, physical activity, tobacco or nicotine exposure, sleep health, body mass index (BMI), blood lipids, blood glucose, and blood pressure. We used Cox proportional hazards models to evaluate associations between genetic risk categories and CVH levels with early-onset CAVS. In Model 1, the genetic risk was adjusted for age at recruitment, sex, ethnicity, assessment center, townsend deprivation index, average annual household income, educational attainment, chronic kidney disease, number of treatments/medications taken, alcohol consumption status, and the first 20 principal components of ancestry; CVH levels were adjusted for the same covariates except assessment center and the principal components of ancestry. In Model 2, we further adjusted for CVH levels in addition to all Model 1 covariates for the genetic risk categories, and for CVH levels, we additionally adjusted for genetic risk categories, assessment center, and the first 20 principal components of ancestry based on model 1. The trend test used the median value of each group instead of the original group. CVH, cardiovascular health; CI, confidence Interval; HR, hazard Ratio; ref, reference; CAVS, calcified aortic valve stenosis.
