# Supplementary material for: Benefits of Better Cardiovascular Health for Calcific Aortic Valve Stenosis Stratified by Polygenic Risk Score
Source: Genomics Proteomics Bioinformatics. 2025 Nov 6;23(5):qzaf099. doi: 10.1093/gpbjnl/qzaf099 (PMC12812169; doi:10.1093/gpbjnl/qzaf099)
Supplement: qzaf099_Supplementary_Data [file qzaf099_supplementary_data.zip › Table S6.docx]

**Table S6 Genetic Correlations between CAVS and five phenotypic traits**

| **Phenotype** | **Source** | **GWAS sample size** | **Genetic correlation** | **SE** | ***P* value** |
| --- | --- | --- | --- | --- | --- |
| Systolic blood pressure | GCST006624 | 757,601 | 0.199 | 0.046 | **1.76E–5** |
| Diastolic blood pressure | GCST006630 | 757,601 | 0.117 | 0.045 | 0.010 |
| Blood mass index | GIANT | 339,224 | 0.267 | 0.041 | **1.03E–10** |
| Cigarettes smoked per day | GCST007459 | 377,334 | 0.225 | 0.047 | **1.68E–6** |
| Fasting glucose | GCST90002232 | 200,622 | 0.011 | 0.053 | 0.841 |

*Note*: All genetic correlations were estimated using LD-score regression. The phenotype for genetic correlation analysis was derived from GWAS summary statistics of CAVS. SE denotes the standard error of the genetic correlation estimate. P values indicate the statistical significance of the observed genetic correlations. CAVS, calcified aortic valve stenosis.
