# Supplementary material for: Benefits of Better Cardiovascular Health for Calcific Aortic Valve Stenosis Stratified by Polygenic Risk Score
Source: Genomics Proteomics Bioinformatics. 2025 Nov 6;23(5):qzaf099. doi: 10.1093/gpbjnl/qzaf099 (PMC12812169; doi:10.1093/gpbjnl/qzaf099)
Supplement: qzaf099_Supplementary_Data [file qzaf099_supplementary_data.zip › Table S7.docx]

**Table S7 RERI and 95% CI for additive interaction between CVH levels and genetic risk (low genetic risk and ideal CVH are the references)**

| **Outcome** | **CVH levels** | **PRS** | | | | |
| --- | --- | --- | --- | --- | --- | --- |
|  |  | **Intermediate** | |  | **High** | |
|  |  | **RERI** | **95%CI** |  | **RERI** | **95%CI** |
| CAVS | Moderate CVH | 0.71 | −0.18, 1.24 |  | 1.47 | −0.11, 3.11 |
|  | Poor CVH | 1.36 | −0.69, 3.27 |  | **3.77** | **1.30, 8.50** |
| Early-onset CAVS | Moderate CVH | 0.72 | −1.09, 1.70 |  | **2.53** | **0.26, 6.52** |
|  | Poor CVH | 1.23 | −3.81, 5.22 |  | 3.51 | −1.91, 13.74 |
| Late-onset CAVS | Moderate CVH | 0.70 | −0.73, 1.61 |  | 1.07 | −1.78, 3.47 |
|  | Poor CVH | 1.44 | −1.59, 4.29 |  | **4.71** | **0.86, 12.77** |

*Note*: Relative excess risk due to interaction (RERI) and its 95% confidence interval were calculated using the MOVER method to assess additive interactions. The Cox models were adjusted for age at recruitment, sex, ethnicity, townsend deprivation index, average annual household income, educational attainment, chronic kidney disease, number of treatments/medications taken, alcohol consumption status, assessment center, and the first 20 principal components of ancestry. Low genetic risk and ideal CVH are the references.
