# Supplementary material for: Benefits of Better Cardiovascular Health for Calcific Aortic Valve Stenosis Stratified by Polygenic Risk Score
Source: Genomics Proteomics Bioinformatics. 2025 Nov 6;23(5):qzaf099. doi: 10.1093/gpbjnl/qzaf099 (PMC12812169; doi:10.1093/gpbjnl/qzaf099)
Supplement: qzaf099_Supplementary_Data [file qzaf099_supplementary_data.zip › Table S9.docx]

**Table S9 Risk of late-onset CAVS (diagnosis age ≥ 65) by joint categorization for genetic risk and CVH levels (*n* = 153,312)**

| **Subgroup** | **Events/Person-years** | **HR (95% CI)** | ***P* value** |
| --- | --- | --- | --- |
| **High genetic risk** | |  |  |
| Poor CVH | 40/21,853 | Ref |  |
| Moderate CVH | 338/308,417 | 0.60 (0.43, 0.84) | **0.003** |
| Ideal CVH | 29/71,736 | 0.42 (0.26, 0.69) | **4.75E–4** |
|  |  |  |  |
| **Intermediate genetic risk** |  |  |  |
| Poor CVH | 48/58,912 | 0.42 (0.28, 0.64) | **5.03E–5** |
| Moderate CVH | 461/918,906 | 0.28 (0.20, 0.38) | **1.53E–14** |
| Ideal CVH | 33/231,699 | 0.15 (0.09, 0.23) | **5.47E–16** |
|  |  |  |  |
| **Low genetic risk** |  |  |  |
| Poor CVH | 8/18,359 | 0.22 (0.11, 0.48) | **1.17E–4** |
| Moderate CVH | 92/303,576 | 0.17 (0.12, 0.24) | **< 2E–16** |
| Ideal CVH | 10/82,233 | 0.12 (0.06, 0.25) | **3.64E–9** |

*Note*: We used Cox proportional hazards model to evaluate the association between combined genetic risk categories and CVH levels and the risk of late-onset CAVS. The model was adjusted for age at recruitment, sex, ethnicity, townsend deprivation index, average annual household income, educational attainment, chronic kidney disease, number of treatments/medications taken, alcohol consumption status, assessment center and first 20 principal components of ancestry. CVH, cardiovascular health; CAVS, calcific aortic valve stenosis; HR, hazard ratio; CI, confidence interval.
