# Supplementary material for: Benefits of Better Cardiovascular Health for Calcific Aortic Valve Stenosis Stratified by Polygenic Risk Score
Source: Genomics Proteomics Bioinformatics. 2025 Nov 6;23(5):qzaf099. doi: 10.1093/gpbjnl/qzaf099 (PMC12812169; doi:10.1093/gpbjnl/qzaf099)
Supplement: qzaf099_Supplementary_Data [file qzaf099_supplementary_data.zip › Table S10.docx]

**Table S10 Risk of early-onset CAVS (diagnosis age < 65) by joint categorization for genetic risk and CVH levels (*n* = 129,066)**

| **Subgroup** | **Events/Person-years** | **HR (95% CI)** | ***P* value** |
| --- | --- | --- | --- |
| **High genetic risk** | |  |  |
| Poor CVH | 12/13,083 | Ref |  |
| Moderate CVH | 71/168,591 | 0.71 (0.38, 1.32) | 0.273 |
| Ideal CVH | 4/50,901 | 0.21 (0.07, 0.68) | **0.009** |
|  |  |  |  |
| **Intermediate genetic risk** |  |  |  |
| Poor CVH | 15/34,069 | 0.48 (0.22, 1.02) | 0.057 |
| Moderate CVH | 77/499,079 | 0.26 (0.14, 0.49) | **2.71E–5** |
| Ideal CVH | 10/160,604 | 0.17 (0.07, 0.39) | **4.61E–5** |
|  |  |  |  |
| **Low genetic risk** |  |  |  |
| Poor CVH | 3/10,630 | 0.29 (0.08, 1.03) | 0.056 |
| Moderate CVH | 15/163,418 | 0.16 (0.07, 0.34) | **2.42E–6** |
| Ideal CVH | 5/56,987 | 0.23 (0.08, 0.67) | **0.007** |

*Note*: We used cox proportional hazards model to evaluate the association between combined genetic risk categories and CVH levels and the risk of early-onset CAVS. The model was adjusted for age at recruitment, sex, ethnicity, townsend deprivation index, average annual household income, educational attainment, chronic kidney disease, number of treatments/medications taken, alcohol consumption status, assessment center and first 20 principal components of ancestry. CVH, cardiovascular health; CAVS, calcific aortic valve stenosis; HR, hazard ratio; CI, confidence interval.
