# Supplementary material for: Benefits of Better Cardiovascular Health for Calcific Aortic Valve Stenosis Stratified by Polygenic Risk Score
Source: Genomics Proteomics Bioinformatics. 2025 Nov 6;23(5):qzaf099. doi: 10.1093/gpbjnl/qzaf099 (PMC12812169; doi:10.1093/gpbjnl/qzaf099)
Supplement: qzaf099_Supplementary_Data [file qzaf099_supplementary_data.zip › Table S11.docx]

**Table S11 Risk of CAVS by joint categorization for genetic risk and CVH levels after excluding incident CAVS within the first 2 years of follow-up (*n* = 153,262)**

| **Subgroup** | **Events/Person-years** | **HR (95% CI)** | ***P* value** |
| --- | --- | --- | --- |
| **High genetic risk** | |  |  |
| Poor CVH | 52/21,853 | Ref |  |
| Moderate CVH | 393/308,401 | 0.59 (0.44, 0.79) | **4.31E–4** |
| Ideal CVH | 33/71,736 | 0.38 (0.24, 0.59) | **1.57E–5** |
|  |  |  |  |
| **Intermediate genetic risk** |  |  |  |
| Poor CVH | 61/58,910 | 0.42 (0.29, 0.60) | **3.42E–6** |
| Moderate CVH | 512/918,881 | 0.26 (0.19, 0.35) | **< 2E–16** |
| Ideal CVH | 42/231,699 | 0.15 (0.10, 0.22) | **< 2E–16** |
|  |  |  |  |
| **Low genetic risk** |  |  |  |
| Poor CVH | 10/18,358 | 0.22 (0.11, 0.43) | **9.27E–6** |
| Moderate CVH | 105/303,573 | 0.16 (0.12, 0.23) | **< 2E–16** |
| Ideal CVH | 13/82,230 | 0.13 (0.07, 0.23) | **3.38E–11** |

*Note*: We used Cox proportional hazards models to evaluate the associations between combined genetic risk categories and CVH levels and the risk of CAVS. The model was adjusted for age at recruitment, sex, ethnicity, townsend deprivation index, average annual household income, educational attainment, chronic kidney disease, number of treatments/medications taken, alcohol consumption status, assessment center and first 20 principal components of ancestry. CVH, cardiovascular health; CAVS, calcific aortic valve stenosis; HR, hazard ratio; CI, confidence interval.
