# Supplementary material for: Benefits of Better Cardiovascular Health for Calcific Aortic Valve Stenosis Stratified by Polygenic Risk Score
Source: Genomics Proteomics Bioinformatics. 2025 Nov 6;23(5):qzaf099. doi: 10.1093/gpbjnl/qzaf099 (PMC12812169; doi:10.1093/gpbjnl/qzaf099)
Supplement: qzaf099_Supplementary_Data [file qzaf099_supplementary_data.zip › Table S13.docx]

**Table S13 Associations between CVH levels and risk of CAVS after adjustment for additional medication use**

| **Subgroup** | **Events/person years** | **HR (95% CI)** | ***P* value** |
| --- | --- | --- | --- |
| **Antiplatelet medication** |  |  |  |
| Ideal CVH | 91/385,669 | Ref |  |
| Moderate CVH | 1054/1,530,899 | 1.64 (1.32, 2.04) | **7.23E–6** |
| Poor CVH | 126/99,123 | 2.57 (1.94, 3.39) | **3.00E–11** |
| P trend |  |  | **1.28E–11** |
|  |  |  |  |
| **Anticoagulant medication** |  |  |  |
| Ideal CVH | 91/385,669 | Ref |  |
| Moderate CVH | 1054/1,530,899 | 1.64 (1.32, 2.04) | **6.85E–6** |
| Poor CVH | 126/99,123 | 2.57 (1.94, 3.39) | **3.09E–11** |
| P trend |  |  | **1.34E–11** |
|  |  |  |  |
| **Antiresorptive medication** |  |  |  |
| Ideal CVH | 91/385,669 | Ref |  |
| Moderate CVH | 1054/1,530,899 | 1.64 (1.32, 2.04) | **7.10E–6** |
| Poor CVH | 126/99,123 | 2.57 (1.94, 3.39) | **3.08E–11** |
| P trend |  |  | **1.32E–11** |
|  |  |  |  |
| **Supplements (vitamin D, vitamin K, calcium supplement)** |  |  |  |
| Ideal CVH | 91/385,669 | Ref |  |
| Moderate CVH | 1054/1,530,899 | 1.64 (1.32, 2.04) | **7.04E–6** |
| Poor CVH | 126/99,123 | 2.57 (1.94, 3.39) | **3.11E–11** |
| P trend |  |  | **1.34E–11** |
|  |  |  |  |
| **Above four medications** |  |  |  |
| Ideal CVH | 91/385,669 | Ref |  |
| Moderate CVH | 1054/1,530,899 | 1.64 (1.32, 2.04) | **7.66E–6** |
| Poor CVH | 126/99,123 | 2.56 (1.94, 3.39) | **3.61E–11** |
| P trend |  |  | **1.58E–11** |

*Note*: We used Cox proportional hazards models to evaluate the associations between CVH levels and the risk of CAVS. The models were adjusted for genetic risk, age at recruitment, sex, ethnicity, townsend deprivation index, average annual household income, educational attainment, chronic kidney disease, number of treatments/medications taken, alcohol consumption status, assessment center and first 20 principal components of ancestry. The models were further adjusted for additional medication use (antiplatelet, anticoagulant, antiresorptive, and vitamin/mineral supplements) both individually and in combination. CVH, cardiovascular health; CAVS, calcific aortic valve stenosis; LE8, Life’s Essential 8; HR, hazard ratio; CI, confidence interval.
