# Supplementary material for: Benefits of Better Cardiovascular Health for Calcific Aortic Valve Stenosis Stratified by Polygenic Risk Score
Source: Genomics Proteomics Bioinformatics. 2025 Nov 6;23(5):qzaf099. doi: 10.1093/gpbjnl/qzaf099 (PMC12812169; doi:10.1093/gpbjnl/qzaf099)
Supplement: qzaf099_Supplementary_Data [file qzaf099_supplementary_data.zip › Table S14.docx]

**Table S14** **Distribution of CAVS events at 3-, 5-, and 10-year follow-up by CVH levels and genetic risk**

|  |  | **Poor CVH (0–49 Points)** | **Moderate CVH (50–79 Points)** | **Ideal CVH (80–100 Points)** |  |
| --- | --- | --- | --- | --- | --- |
| N |  | 7741 | 116,668 | 28,903 |  |
| Age, years |  | 56.25 ± 7.40 | 56.65 ± 7.82 | 53.13 ± 8.05 |  |
| Follow-up duration (years), (median [IQR]) |  | 13.15 (12.56, 13.96) | 13.26 (12.65, 14.04) | 13.35 (12.74, 14.13) |  |
|  |  |  |  |  |  |
| Lifelong cumulative incidence, No. (%) |  |  |  |  |  |
|  | CAVS | 126 (1.63) | 1,054 (0.90) | 91 (0.31) |  |
| Stratified by genetic risk  (Cases) |  |  |  |  |  |
|  | High (top 20%) | 52 (0.67) | 409 (0.35) | 33 (0.11) |  |
|  | Intermediate (middle 60%) | 63 (0.81) | 538 (0.46) | 43 (0.15) |  |
|  | Low (bottom 20%) | 11 (0.14) | 107 (0.09) | 15 (0.05) |  |
|  |  |  |  |  |  |
| 3-year cumulative incidence, No. (%) |  |  |  |  |  |
|  | CAVS | 7 (0.09) | 78 (0.07) | 4 (0.01) |  |
| Stratified by genetic risk  (3-year follow-up, Cases) |  |  |  |  |  |
|  | High (top 20%) | 1 (0.01) | 29 (0.02) | 1 (0.00) |  |
|  | Intermediate (middle 60%) | 5 (0.06) | 41 (0.04) | 1 (0.00) |  |
|  | Low (bottom 20%) | 1 (0.01) | 8 (0.01) | 2 (0.01) |  |
|  |  |  |  |  |  |
| 5-year cumulative incidence, No. (%) |  |  |  |  |  |
|  | CAVS | 24 (0.31) | 170 (0.15) | 10 (0.03) |  |
| Stratified by genetic risk  (5-year follow-up, Cases) |  |  |  |  |  |
|  | High (top 20%) | 5 (0.06) | 64 (0.05) | 5 (0.02) |  |
|  | Intermediate (middle 60%) | 16 (0.21) | 91 (0.08) | 1 (0.00) |  |
|  | Low (bottom 20%) | 3 (0.04) | 15 (0.01) | 4 (0.01) |  |
|  |  |  |  |  |  |
| 10-year cumulative incidence, No. (%) |  |  |  |  |  |
|  | CAVS | 83 (1.07) | 580 (0.50) | 48 (0.17) |  |
| Stratified by genetic risk  (10-year follow-up, Cases) |  |  |  |  |  |
|  | High (top 20%) | 31 (0.40) | 236 (0.20) | 18 (0.06) |  |
|  | Intermediate (middle 60%) | 44 (0.57) | 289 (0.25) | 24 (0.08) |  |
|  | Low (bottom 20%) | 8 (0.10) | 55 (0.05) | 6 (0.02) |  |

*Note*: Continuous variables are presented by as mean (SD) or median (interquartile range). Categorical variables are presented as N (%). Data are presented as the number of CAVS cases observed at 3-, 5-, and 10-year follow-up, overall and stratified by CVH level (based on LE8 score) and genetic risk category. Percentages in parentheses represent the proportion of events relative to the total number of participants within each CVH category. CVH, cardiovascular health; LE8, Life’s Essential 8; CAVS, calcific aortic valve stenosis; IQR, interquartile range; SD, standard deviation.
