# Supplementary material for: Benefits of Better Cardiovascular Health for Calcific Aortic Valve Stenosis Stratified by Polygenic Risk Score
Source: Genomics Proteomics Bioinformatics. 2025 Nov 6;23(5):qzaf099. doi: 10.1093/gpbjnl/qzaf099 (PMC12812169; doi:10.1093/gpbjnl/qzaf099)
Supplement: qzaf099_Supplementary_Data [file qzaf099_supplementary_data.zip › Table S15.docx]

**Table S15** **Sex-stratified analyses of the associations between each LE8 metric and the risk of CAVS in the overall population after adjusting genetic risk**

| **Lifestyle factors** | **Events/person years** | **HR/SD (95% CI)** | ***P* value** | ***P* for interaction** |
| --- | --- | --- | --- | --- |
| **Blood pressure score** | 1271/2,015,691 | **0.84 (0.78, 0.89)** | **1.96E–7** | 0.123 |
| Female | 418/1,086,263 | **0.80 (0.72, 0.90)** | **1.16E–4** |  |
| Male | 853/929,428 | **0.86 (0.79, 0.94)** | **4.56E–4** |  |
|  |  |  |  |  |
| **Body mass index score** | 1271/2,015,691 | **0.80 (0.76, 0.85)** | **1.09E–13** | 0.216 |
| Female | 418/1,086,263 | **0.80 (0.73, 0.87)** | **1.33E–6** |  |
| Male | 853/929,428 | **0.81 (0.75, 0.87)** | **3.02E–8** |  |
|  |  |  |  |  |
| **Tobacco/nicotine exposure score** | 1271/2,015,691 | **0.89 (0.84, 0.95)** | **9.09E–5** | 0.168 |
| Female | **418/1,086,263** | **0.85 (0.77, 0.94)** | **0.001** |  |
| Male | 853/929,428 | **0.91 (0.85, 0.98)** | **0.010** |  |
|  |  |  |  |  |
| **Blood glucose score** | 1271/2,015,691 | **0.89 (0.85, 0.93)** | **1.01E–7** | 0.266 |
| Female | 418/1,086,263 | **0.88 (0.81, 0.95)** | **0.002** |  |
| Male | 853/929,428 | **0.89 (0.84, 0.94)** | **1.24E–5** |  |
|  |  |  |  |  |
| **Blood lipid score** | 1271/2,015,691 | **0.87 (0.82, 0.93)** | **1.13E–5** | 0.213 |
| Female | 418/1,086,263 | **0.82 (0.74, 0.91)** | **2.78E–4** |  |
| Male | 853/929,428 | 0.91 (0.84, 0.98) | 0.011 |  |
|  |  |  |  |  |
| **DASH score** | 1271/2,015,691 | 0.99 (0.93, 1.05) | 0.719 | 0.900 |
| Female | 418/1,086,263 | 1.01 (0.91, 1.11) | 0.870 |  |
| Male | 853/929,428 | 0.98 (0.92, 1.06) | 0.674 |  |
|  |  |  |  |  |
| **Physical activity score** | 1271/2,015,691 | 1.00 (0.95, 1.06) | 0.806 | 0.986 |
| Female | 418/1,086,263 | 1.02 (0.92, 1.12) | 0.725 |  |
| Male | 853/929,428 | 1.00 (0.94, 1.07) | 0.998 |  |
|  |  |  |  |  |
| **Sleep health score** | 1271/2,015,691 | 1.00 (0.95, 1.06) | 0.875 | **0.023** |
| Female | 418/1,086,263 | 0.94 (0.87, 1.02) | 0.166 |  |
| Male | 853/929,428 | 1.05 (0.97, 1.12) | 0.216 |  |

*Note*: We used cox proportional hazards models to evaluate associations between each LE8 metric with CAVS, with all eight LE8 metrics simultaneously included in the multivariable-adjusted models. The models were adjusted for genetic risk, age at recruitment, sex, ethnicity, Townsend deprivation index, average annual household income, educational attainment, chronic kidney disease, number of treatments/medications taken, alcohol consumption status, assessment center, and the first 20 principal components of ancestry. Sex-stratified analyses were performed on the association between individual LE8 metrics with CAVS risk and to explore potential multiplicative interactions between each LE8 metric and CAVS risk within male and female subgroups, respectively. Bonferroni-corrected significance threshold was applied for 8 lifestyle components (P < 0.05/8 = 0.00625). CAVS, calcific aortic valve stenosis; CI, confidence interval; HR, hazard ratio; LE8, Life’s Essential 8; CVH, cardiovascular health; DASH, Dietary Approaches to Stop Hypertension; TDI, Townsend deprivation index; Ref, reference.
