# Supplementary material for: Benefits of Better Cardiovascular Health for Calcific Aortic Valve Stenosis Stratified by Polygenic Risk Score
Source: Genomics Proteomics Bioinformatics. 2025 Nov 6;23(5):qzaf099. doi: 10.1093/gpbjnl/qzaf099 (PMC12812169; doi:10.1093/gpbjnl/qzaf099)
Supplement: qzaf099_Supplementary_Data [file qzaf099_supplementary_data.zip › Table S17.docx]

**Table S17 Sex-stratified associations between genetic risk (LDpred2-derived PRS based on MVP GWAS summary) with early-onset and late-onset CAVS**

| **Outcome** | **Subgroup** | **Events/person years** | **Model 1 HR/SD (95% CI)** | ***P*** | **Model 2 HR/SD (95% CI)** | ***P*** | ***P* for interaction** |
| --- | --- | --- | --- | --- | --- | --- | --- |
| **Early-onset CAVS** | **Male** |  |  |  |  |  | 0.091 |
|  | Genetic risk score | 146/507,064 | 1.59 (1.35, 1.87) | **1.82E–8** | 1.59 (1.35, 1.86) | **2.34E–8** |  |
|  | **Female** |  |  |  |  |  |  |
|  | Genetic risk score | 66/650,299 | 2.03 (1.60, 2.58) | **6.78E–9** | 2.02 (1.59, 2.56) | **9.59E–9** |  |
| **Late-onset CAVS** | **Male** |  |  |  |  |  | 0.207 |
|  | Genetic risk score | 707/929,428 | 1.67 (1.55, 1.80) | **< 2E–16** | 1.66 (1.55, 1.79) | **< 2E–16** |  |
|  | **Female** |  |  |  |  |  |  |
|  | Genetic risk score | 352/1,086,263 | 1.54 (1.38, 1.70) | **5.20E–16** | 1.53 (1.38, 1.70) | **1.08E–15** |  |

*Note*: Genetic risk categories were defined using an LDpred2-derived polygenic risk score. Sex-stratified subgroup analyses were performed to evaluate the associations between genetic risk and risks of early-onset and late**-**onset CAVS separately in male and female participants using cox proportional hazards models. In Model 1, The models were adjusted for age at recruitment, ethnicity, assessment center, Townsend deprivation index, average annual household income, educational attainment, chronic kidney disease, number of treatments/medications taken, alcohol consumption status, and the first 20 principal components of ancestry. Model 2 was further adjusted for CVH levels based on the Life’s Essential 8 (LE8) score. P for interaction was derived from the multiplicative interaction term between sex and genetic risk score. CVH, cardiovascular health; CI, confidence interval; HR, hazard ratio; PRS, polygenic risk score; ref, reference.
