# Supplementary material for: Benefits of Better Cardiovascular Health for Calcific Aortic Valve Stenosis Stratified by Polygenic Risk Score
Source: Genomics Proteomics Bioinformatics. 2025 Nov 6;23(5):qzaf099. doi: 10.1093/gpbjnl/qzaf099 (PMC12812169; doi:10.1093/gpbjnl/qzaf099)
Supplement: qzaf099_Supplementary_Data [file qzaf099_supplementary_data.zip › Table S18.docx]

**Table S18 Distribution of CAVS events across ethnic groups in the included cohort (*n* = 153,312)**

|  |  | **CAVS** | **Control** |
| --- | --- | --- | --- |
| N |  | 1271 | 152,041 |
| Ethnicity, No. (%) | White | 1251 (98.43) | 145,866 (95.94) |
|  | Mixed | 3 (0.24) | 861 (0.57) |
|  | Asian | 9 (0.71) | 1967 (1.29) |
|  | Black | 2 (0.15) | 1479 (0.97) |
|  | Chinese | 0 (0.00) | 417 (0.27) |
|  | Other ethnic group | 3 (0.24) | 1015 (0.67) |
|  | Unknown/missing | 3 (0.24) | 436 (0.29) |

*Note*: This table shows the distribution of CAVS events across self-reported ethnic groups in the Included Cohort. Categorical variables are presented as N (%). CAVS, calcified aortic valve stenosis.
