# Supplementary material for: Benefits of Better Cardiovascular Health for Calcific Aortic Valve Stenosis Stratified by Polygenic Risk Score
Source: Genomics Proteomics Bioinformatics. 2025 Nov 6;23(5):qzaf099. doi: 10.1093/gpbjnl/qzaf099 (PMC12812169; doi:10.1093/gpbjnl/qzaf099)
Supplement: qzaf099_Supplementary_Data [file qzaf099_supplementary_data.zip › Table S19.docx]

**Table S19 Single-nucleotide polymorphisms used to build the genetic risk score including 29 SNPs (*P* ≤ 5 × 10^‒8^)**

| **SNP** | **Chr** | **Pos_hg37** | **ALT** | **REF** | **Beta** | ***P* value** |
| --- | --- | --- | --- | --- | --- | --- |
| rs6702619 | 1 | 100046246 | T | G | −0.13 | 7.80E−35 |
| rs4970836 | 1 | 109821797 | G | A | −0.08 | 4.24E−10 |
| rs61817383 | 1 | 170665348 | C | T | −0.07 | 8.96E−09 |
| rs12118362 | 1 | 21771997 | G | A | −0.08 | 3.32E−08 |
| rs10186882 | 2 | 146344233 | A | G | −0.08 | 1.56E−08 |
| rs1505373 | 2 | 213242684 | C | T | 0.06 | 1.36E−08 |
| rs62139061 | 2 | 65498805 | T | C | −0.07 | 4.82E−09 |
| rs71630059 | 3 | 136581611 | G | A | 0.08 | 2.38E−09 |
| rs2421651 | 3 | 169206493 | A | G | −0.08 | 2.42E−14 |
| rs1706003 | 3 | 194299967 | G | T | 0.06 | 1.24E−08 |
| rs9831340 | 3 | 57953782 | C | A | −0.06 | 7.72E−10 |
| rs11153733 | 6 | 118724727 | T | C | −0.17 | 7.03E−13 |
| rs12527112 | 6 | 132091560 | A | G | −0.10 | 4.70E−08 |
| rs74617384 | 6 | 160997118 | A | T | −0.34 | 2.44E−65 |
| rs73596816 | 6 | 161017363 | G | A | −0.16 | 1.97E−09 |
| rs186696265 | 6 | 161111700 | C | T | −0.42 | 7.20E−22 |
| rs2106550 | 7 | 22588412 | A | G | −0.07 | 5.36E−11 |
| rs1554606 | 7 | 22768707 | T | G | 0.09 | 8.55E−20 |
| rs12719037 | 7 | 51208953 | T | C | 0.06 | 4.48E−09 |
| rs111715417 | 8 | 11831078 | A | C | −0.15 | 2.33E−09 |
| rs7905980 | 10 | 17059622 | A | T | −0.06 | 1.43E−08 |
| rs12280388 | 11 | 121670712 | T | C | −0.11 | 8.26E−09 |
| rs174551 | 11 | 61573684 | T | C | 0.10 | 3.08E−19 |
| rs10846742 | 12 | 125308682 | G | A | 0.09 | 3.64E−10 |
| rs112840248 | 15 | 42950885 | C | T | 0.08 | 1.54E−10 |
| rs4243085 | 15 | 79057723 | G | C | 0.07 | 4.78E−09 |
| rs56094641 | 16 | 53806453 | A | G | −0.07 | 5.77E−12 |
| rs7187 | 19 | 11275258 | A | G | 0.06 | 4.88E−08 |
| rs4821688 | 22 | 38105810 | C | T | −0.07 | 4.01E−10 |

*Note*: SNP, single nucleotide polymorphism; Chr, chromosome; ALT, alternative allele; REF, reference allele; Beta, effect size estimate from GWAS summary statistics.
