# Supplementary material for: Benefits of Better Cardiovascular Health for Calcific Aortic Valve Stenosis Stratified by Polygenic Risk Score
Source: Genomics Proteomics Bioinformatics. 2025 Nov 6;23(5):qzaf099. doi: 10.1093/gpbjnl/qzaf099 (PMC12812169; doi:10.1093/gpbjnl/qzaf099)
Supplement: qzaf099_Supplementary_Data [file qzaf099_supplementary_data.zip › Table S20.docx]

**Table S20 Single-nucleotide polymorphisms used to build the genetic risk score including 304 SNPs (*P* ≤ 1 × 10^‒4^)**

| **SNP** | **Chr** | **Pos_hg37** | **ALT** | **REF** | **Beta** | ***P* value** |
| --- | --- | --- | --- | --- | --- | --- |
| rs6659166 | 1 | 15348422 | A | G | −0.05 | 9.44E−05 |
| rs34426856 | 1 | 16006315 | C | T | 0.05 | 4.31E−05 |
| rs114817034 | 1 | 18721216 | G | A | −0.14 | 6.96E−05 |
| rs12118362 | 1 | 21771997 | G | A | −0.08 | 3.32E−08 |
| rs12027423 | 1 | 21854503 | T | C | 0.05 | 1.80E−05 |
| rs55722102 | 1 | 21922132 | T | C | −0.06 | 5.05E−05 |
| rs812490 | 1 | 44271656 | T | C | 0.04 | 6.24E−05 |
| rs4927026 | 1 | 54140843 | A | G | 0.06 | 5.41E−05 |
| rs11591147 | 1 | 55505647 | G | T | 0.26 | 2.12E−06 |
| rs12122154 | 1 | 85775865 | C | T | −0.12 | 2.65E−06 |
| rs1572482 | 1 | 87932256 | C | T | 0.04 | 5.57E−05 |
| rs116454974 | 1 | 99800529 | C | T | 0.20 | 2.26E−05 |
| rs6702619 | 1 | 100046246 | T | G | −0.13 | 7.80E−35 |
| rs4970836 | 1 | 109821797 | G | A | −0.08 | 4.24E−10 |
| rs7527662 | 1 | 113278578 | C | G | 0.05 | 2.82E−05 |
| rs12048493 | 1 | 149927034 | A | C | −0.06 | 9.13E−07 |
| rs150107452 | 1 | 154979973 | C | T | −0.15 | 3.40E−05 |
| rs61817383 | 1 | 170665348 | C | T | −0.07 | 8.96E−09 |
| rs150266749 | 1 | 174506128 | T | C | −0.22 | 6.23E−05 |
| rs146441739 | 1 | 196908385 | C | T | −0.06 | 4.56E−06 |
| rs631556 | 1 | 201743185 | G | A | −0.05 | 1.63E−05 |
| rs4845007 | 1 | 210540254 | G | T | 0.06 | 3.84E−06 |
| rs1393391 | 1 | 217356246 | C | T | −0.07 | 1.42E−05 |
| rs1857105 | 1 | 219272930 | G | A | 0.05 | 4.71E−05 |
| rs6604730 | 1 | 224049363 | G | A | 0.08 | 4.76E−06 |
| rs2770371 | 1 | 234830361 | T | C | −0.05 | 2.55E−05 |
| rs487738 | 1 | 234840803 | A | G | −0.06 | 3.05E−05 |
| rs10754581 | 1 | 236868521 | A | T | 0.09 | 8.44E−06 |
| rs10926995 | 1 | 243502623 | A | G | −0.04 | 3.88E−05 |
| rs116561762 | 2 | 1836239 | G | A | −0.23 | 2.81E−05 |
| rs4669127 | 2 | 7160439 | G | A | −0.06 | 1.33E−06 |
| rs56658728 | 2 | 11954498 | A | G | 0.06 | 2.97E−05 |
| rs6710671 | 2 | 20616425 | C | T | −0.06 | 5.53E−08 |
| rs148817038 | 2 | 26015755 | G | A | −0.19 | 5.04E−05 |
| rs1275983 | 2 | 26910556 | T | C | 0.05 | 4.16E−05 |
| rs77843342 | 2 | 31346270 | T | C | −0.19 | 6.50E−05 |
| rs13428289 | 2 | 37723885 | T | G | 0.15 | 5.32E−05 |
| rs67459735 | 2 | 41964627 | A | G | −0.05 | 1.01E−05 |
| rs62139061 | 2 | 65498805 | T | C | −0.07 | 4.82E−09 |
| rs200137681 | 2 | 65593439 | A | C | 0.07 | 2.21E−05 |
| rs10169824 | 2 | 71805522 | C | T | −0.04 | 3.85E−05 |
| rs17008584 | 2 | 73232809 | G | A | −0.11 | 3.43E−05 |
| rs4522662 | 2 | 136981332 | G | A | −0.06 | 9.81E−05 |
| rs6740731 | 2 | 145270592 | G | A | −0.07 | 9.04E−08 |
| rs10186882 | 2 | 146344233 | A | G | −0.08 | 1.56E−08 |
| rs7608743 | 2 | 150830512 | T | C | 0.04 | 7.06E−05 |
| rs13035934 | 2 | 165073072 | G | A | 0.05 | 4.21E−05 |
| rs6709514 | 2 | 173079815 | G | A | −0.06 | 8.32E−05 |
| rs2366911 | 2 | 179693672 | T | C | −0.05 | 3.98E−06 |
| rs1990258 | 2 | 191675900 | T | C | −0.05 | 5.64E−05 |
| rs1505373 | 2 | 213242684 | C | T | 0.06 | 1.36E−08 |
| rs62186571 | 2 | 214038673 | C | G | −0.04 | 6.85E−05 |
| rs114074340 | 2 | 214995446 | A | G | 0.15 | 9.85E−05 |
| rs7568906 | 2 | 230252609 | T | G | 0.06 | 7.06E−05 |
| rs144882445 | 3 | 3922821 | G | A | 0.40 | 7.41E−05 |
| rs6805868 | 3 | 9552350 | G | A | 0.05 | 4.50E−05 |
| rs4684068 | 3 | 11437513 | A | G | 0.05 | 8.86E−05 |
| rs9830504 | 3 | 25504662 | C | T | −0.08 | 1.43E−05 |
| rs111410234 | 3 | 46460675 | C | T | 0.06 | 6.31E−05 |
| rs66782572 | 3 | 52567617 | A | G | −0.04 | 2.18E−05 |
| rs139881228 | 3 | 55348426 | A | G | −0.19 | 1.29E−05 |
| rs9831340 | 3 | 57953782 | C | A | −0.06 | 7.72E−10 |
| rs9862417 | 3 | 63792668 | C | T | −0.04 | 8.41E−05 |
| rs9841988 | 3 | 66382577 | A | G | 0.07 | 2.47E−05 |
| rs77449114 | 3 | 89690994 | T | G | 0.10 | 7.27E−05 |
| rs7614648 | 3 | 104599438 | G | A | −0.04 | 1.99E−05 |
| rs2008627 | 3 | 123978407 | G | A | 0.05 | 7.25E−06 |
| rs16859047 | 3 | 129108935 | C | T | −0.06 | 8.17E−05 |
| rs71630059 | 3 | 136581611 | G | A | 0.08 | 2.38E−09 |
| rs6787361 | 3 | 153766298 | G | A | 0.06 | 1.46E−06 |
| rs4062228 | 3 | 157470885 | C | T | −0.05 | 7.16E−05 |
| rs17485157 | 3 | 169122837 | G | A | −0.05 | 3.15E−05 |
| rs2421651 | 3 | 169206493 | A | G | −0.08 | 2.42E−14 |
| rs16832402 | 3 | 180999947 | C | G | 0.15 | 1.75E−05 |
| rs9852084 | 3 | 187066179 | G | C | −0.05 | 2.78E−05 |
| rs1706003 | 3 | 194299967 | G | T | 0.06 | 1.24E−08 |
| rs11914982 | 3 | 194408716 | G | C | −0.11 | 5.40E−05 |
| rs2293232 | 3 | 195497143 | C | T | −0.06 | 6.52E−05 |
| rs28485535 | 4 | 7854170 | T | C | −0.06 | 2.32E−07 |
| rs150816050 | 4 | 8339155 | T | A | 0.27 | 8.61E−05 |
| rs71605357 | 4 | 19300150 | A | G | −0.05 | 8.70E−06 |
| rs11731707 | 4 | 23855382 | A | G | −0.05 | 4.56E−05 |
| rs34303987 | 4 | 44135573 | T | A | 0.09 | 3.14E−05 |
| rs2605273 | 4 | 48972768 | T | C | 0.05 | 9.09E−06 |
| rs111347103 | 4 | 54746836 | A | C | −0.06 | 8.59E−06 |
| rs6811340 | 4 | 69452973 | G | C | 0.06 | 5.13E−06 |
| rs12710823 | 4 | 86718850 | A | G | 0.05 | 1.29E−05 |
| rs7439032 | 4 | 86931091 | T | C | 0.06 | 5.77E−06 |
| rs13107325 | 4 | 103188709 | C | T | 0.08 | 3.12E−05 |
| rs35653213 | 4 | 109133777 | A | C | 0.06 | 6.88E−08 |
| rs17039028 | 4 | 109346722 | A | G | 0.08 | 2.03E−05 |
| rs144068684 | 4 | 120429672 | C | A | −0.27 | 6.32E−06 |
| rs73848317 | 4 | 137836028 | G | A | −0.05 | 9.49E−05 |
| rs74490334 | 4 | 167477364 | C | T | 0.06 | 5.50E−05 |
| rs454190 | 5 | 694413 | T | C | 0.07 | 4.47E−05 |
| rs74506114 | 5 | 11830074 | T | C | −0.18 | 5.10E−06 |
| rs445611 | 5 | 35396119 | A | G | −0.07 | 3.13E−07 |
| rs116623574 | 5 | 52023538 | G | A | −0.18 | 3.61E−05 |
| rs6885424 | 5 | 52177064 | T | C | 0.13 | 7.19E−05 |
| rs80131293 | 5 | 64150534 | C | T | −0.08 | 5.12E−05 |
| rs10515077 | 5 | 67613801 | A | C | −0.07 | 8.57E−05 |
| rs13153194 | 5 | 73365374 | T | C | 0.08 | 3.23E−05 |
| rs35350697 | 5 | 81688577 | C | T | −0.07 | 3.39E−05 |
| rs76811595 | 5 | 81941314 | C | A | −0.08 | 9.09E−05 |
| rs16900386 | 5 | 82686764 | T | C | −0.11 | 6.97E−05 |
| rs548748399 | 5 | 108163543 | C | T | 0.07 | 8.99E−05 |
| rs876600 | 5 | 128235848 | T | A | −0.04 | 4.61E−05 |
| rs199966301 | 5 | 132469684 | A | C | −0.12 | 4.13E−05 |
| rs6867761 | 5 | 135977572 | G | C | −0.04 | 5.91E−05 |
| rs145587240 | 5 | 138046844 | C | T | 0.19 | 6.30E−06 |
| rs1368298 | 5 | 158204425 | A | G | 0.05 | 3.57E−06 |
| rs547327 | 5 | 172320128 | T | C | 0.04 | 6.61E−05 |
| rs7356620 | 5 | 172644520 | A | G | 0.05 | 9.48E−05 |
| rs76139088 | 5 | 174302329 | A | G | −0.20 | 3.28E−05 |
| rs72823216 | 6 | 6744700 | G | A | 0.08 | 6.61E−05 |
| rs3134952 | 6 | 32113571 | A | T | −0.07 | 1.69E−06 |
| rs6910300 | 6 | 35534371 | A | G | 0.05 | 2.12E−05 |
| rs11153733 | 6 | 118724727 | T | C | −0.17 | 7.03E−13 |
| rs9402217 | 6 | 130466234 | G | A | −0.04 | 8.69E−05 |
| rs12527112 | 6 | 132091560 | A | G | −0.10 | 4.70E−08 |
| rs2038551 | 6 | 136059177 | G | T | −0.06 | 1.44E−07 |
| rs12530095 | 6 | 140738349 | G | A | 0.05 | 2.43E−05 |
| rs4895588 | 6 | 141497409 | G | A | −0.05 | 3.39E−05 |
| rs77009034 | 6 | 155354695 | C | T | −0.12 | 6.90E−05 |
| rs74617384 | 6 | 160997118 | A | T | −0.34 | 2.44E−65 |
| rs73596816 | 6 | 161017363 | G | A | −0.16 | 1.97E−09 |
| rs11751347 | 6 | 161092438 | C | T | 0.09 | 8.84E−06 |
| rs570900614 | 6 | 161103459 | A | C | 0.14 | 1.02E−05 |
| rs186696265 | 6 | 161111700 | C | T | −0.42 | 7.20E−22 |
| rs1247339 | 6 | 161375089 | A | G | 0.05 | 6.25E−05 |
| rs6926283 | 6 | 170734451 | T | C | −0.04 | 2.71E−05 |
| rs9330400 | 7 | 588423 | T | A | −0.04 | 4.44E−05 |
| rs62434489 | 7 | 7865239 | T | G | 0.07 | 8.65E−05 |
| rs6967913 | 7 | 11834671 | A | G | −0.06 | 6.76E−06 |
| rs12154315 | 7 | 14373714 | C | T | −0.05 | 9.44E−06 |
| rs2192473 | 7 | 19464027 | A | G | 0.06 | 4.59E−07 |
| rs2106550 | 7 | 22588412 | A | G | −0.07 | 5.36E−11 |
| rs1554606 | 7 | 22768707 | T | G | 0.09 | 8.55E−20 |
| rs6956175 | 7 | 28904493 | A | G | 0.05 | 5.20E−05 |
| rs886834 | 7 | 29021336 | G | A | −0.05 | 7.31E−05 |
| rs17172071 | 7 | 42523690 | G | A | 0.06 | 2.49E−06 |
| rs12719037 | 7 | 51208953 | T | C | 0.06 | 4.48E−09 |
| rs2260084 | 7 | 76635006 | C | T | 0.06 | 6.06E−05 |
| rs2372800 | 7 | 85205095 | T | G | −0.05 | 1.04E−05 |
| rs7786226 | 7 | 92254856 | C | A | 0.06 | 5.10E−08 |
| rs62466352 | 7 | 101347576 | A | G | 0.09 | 1.29E−05 |
| rs201906653 | 7 | 106386361 | C | T | −0.07 | 4.55E−05 |
| rs193686 | 7 | 116431427 | C | T | 0.05 | 1.01E−05 |
| rs112890698 | 7 | 123425226 | G | A | −0.17 | 3.64E−05 |
| rs3800685 | 7 | 131192428 | G | A | 0.05 | 9.83E−05 |
| rs112485170 | 7 | 148275317 | C | T | −0.14 | 4.63E−05 |
| rs188241113 | 7 | 157821876 | A | T | 0.15 | 9.09E−05 |
| rs77626393 | 8 | 1193208 | G | A | −0.14 | 4.40E−05 |
| rs117483167 | 8 | 4990847 | T | A | −0.22 | 8.87E−06 |
| rs60783994 | 8 | 5499927 | C | T | 0.12 | 5.62E−05 |
| rs2264309 | 8 | 11510275 | C | G | 0.06 | 3.28E−05 |
| rs3757949 | 8 | 11614998 | G | C | −0.06 | 3.96E−06 |
| rs111715417 | 8 | 11831078 | A | C | −0.15 | 2.33E−09 |
| rs115849089 | 8 | 19912370 | G | A | 0.07 | 3.84E−05 |
| rs2978462 | 8 | 23403594 | G | A | −0.14 | 3.97E−05 |
| rs118031766 | 8 | 62172701 | A | G | −0.23 | 8.65E−05 |
| rs12216772 | 8 | 106573919 | C | T | −0.05 | 6.48E−07 |
| rs6986073 | 8 | 116048790 | G | C | −0.11 | 8.15E−06 |
| rs13280592 | 8 | 116686752 | C | G | −0.05 | 2.30E−05 |
| rs6982502 | 8 | 126479362 | C | T | 0.06 | 1.02E−07 |
| rs45613837 | 8 | 141744917 | G | A | −0.05 | 1.01E−06 |
| rs117431711 | 8 | 143389945 | G | A | −0.23 | 4.22E−05 |
| rs9407764 | 9 | 16377360 | T | C | 0.04 | 7.64E−05 |
| rs1416511 | 9 | 23104543 | C | G | 0.07 | 3.22E−05 |
| rs117298470 | 9 | 26084236 | A | G | 0.16 | 1.68E−05 |
| rs944584 | 9 | 32458940 | C | T | 0.05 | 1.28E−05 |
| rs7022500 | 9 | 82858862 | T | C | 0.04 | 5.66E−05 |
| rs55714459 | 9 | 96434411 | C | T | −0.06 | 4.49E−07 |
| rs7350148 | 9 | 106883085 | T | G | −0.05 | 3.68E−06 |
| rs7020341 | 9 | 119247974 | G | C | −0.05 | 2.02E−05 |
| rs35503918 | 9 | 123503887 | T | C | 0.06 | 3.79E−05 |
| rs2798433 | 9 | 130293423 | C | T | 0.05 | 1.07E−06 |
| rs6478960 | 9 | 133070510 | A | G | 0.05 | 4.35E−05 |
| rs3892282 | 9 | 133419328 | G | T | −0.04 | 7.00E−05 |
| rs10993806 | 9 | 136455749 | C | T | 0.05 | 5.77E−05 |
| rs148167113 | 9 | 139369066 | G | A | 0.22 | 3.48E−06 |
| rs79268857 | 10 | 5528448 | G | A | 0.40 | 7.34E−05 |
| rs7905980 | 10 | 17059622 | A | T | −0.06 | 1.43E−08 |
| rs11007785 | 10 | 30153125 | A | C | −0.06 | 3.81E−06 |
| rs1778871 | 10 | 34535021 | C | G | −0.06 | 7.97E−05 |
| rs117255603 | 10 | 60814045 | T | C | −0.52 | 7.95E−06 |
| rs224111 | 10 | 64552010 | G | A | −0.04 | 8.34E−05 |
| rs730334 | 10 | 73758332 | C | T | −0.05 | 1.89E−06 |
| rs137866511 | 10 | 75913095 | G | A | −0.15 | 7.76E−06 |
| rs11597938 | 10 | 78685670 | G | A | 0.17 | 8.18E−05 |
| rs11202155 | 10 | 88511321 | C | T | 0.04 | 7.55E−05 |
| rs147027904 | 10 | 93282685 | A | C | 0.24 | 7.55E−06 |
| rs1223618 | 10 | 95777231 | T | C | 0.05 | 3.51E−06 |
| rs113226859 | 10 | 108442229 | G | A | 0.26 | 7.30E−05 |
| rs12774592 | 10 | 120876844 | G | A | 0.05 | 3.29E−06 |
| rs12217849 | 10 | 124930770 | A | G | 0.07 | 7.46E−06 |
| rs28706163 | 11 | 196573 | A | G | 0.09 | 9.44E−07 |
| rs12283151 | 11 | 19033096 | C | T | 0.18 | 2.28E−06 |
| rs1462544 | 11 | 23536577 | G | A | −0.04 | 7.21E−05 |
| rs12807253 | 11 | 27628269 | G | A | 0.08 | 8.18E−05 |
| rs174551 | 11 | 61573684 | T | C | 0.10 | 3.08E−19 |
| rs12796984 | 11 | 66735215 | G | A | −0.11 | 1.15E−05 |
| rs544188596 | 11 | 71738468 | C | A | −0.11 | 7.72E−05 |
| rs2446117 | 11 | 81015307 | C | T | 0.06 | 5.28E−06 |
| rs76330885 | 11 | 84535701 | A | C | −0.10 | 2.81E−06 |
| rs11020158 | 11 | 92806857 | T | A | 0.15 | 6.05E−05 |
| rs150544590 | 11 | 113695035 | G | C | −0.21 | 5.82E−05 |
| rs7115855 | 11 | 115464938 | T | A | 0.04 | 8.47E−05 |
| rs11604424 | 11 | 116651115 | C | T | 0.05 | 7.86E−05 |
| rs148400182 | 11 | 118681325 | A | C | −0.16 | 1.62E−05 |
| rs12280388 | 11 | 121670712 | T | C | −0.11 | 8.26E−09 |
| rs588361 | 11 | 126084834 | G | A | 0.05 | 4.32E−06 |
| rs7974933 | 12 | 1513912 | A | G | 0.08 | 4.31E−05 |
| rs61909253 | 12 | 4486888 | A | G | −0.08 | 3.56E−07 |
| rs6489546 | 12 | 4562344 | A | G | −0.06 | 1.03E−06 |
| rs76604460 | 12 | 11761871 | A | G | 0.20 | 6.94E−05 |
| rs4763751 | 12 | 12167729 | C | A | −0.06 | 6.96E−05 |
| rs10770612 | 12 | 20230639 | A | G | 0.06 | 1.13E−05 |
| rs487165 | 12 | 50304588 | A | T | −0.05 | 3.22E−05 |
| rs10784475 | 12 | 65971563 | T | A | 0.06 | 4.63E−05 |
| rs141753469 | 12 | 66397166 | A | C | 0.07 | 8.18E−07 |
| rs11116859 | 12 | 85927862 | C | T | −0.07 | 1.54E−05 |
| rs6538455 | 12 | 94221094 | A | G | 0.05 | 3.76E−05 |
| rs73372215 | 12 | 94849107 | C | T | 0.08 | 5.05E−05 |
| rs77401548 | 12 | 95989561 | A | G | −0.11 | 3.25E−06 |
| rs10774873 | 12 | 110181131 | C | T | 0.04 | 6.13E−05 |
| rs7952823 | 12 | 110625149 | G | A | 0.05 | 1.06E−05 |
| rs11067104 | 12 | 114854089 | G | C | −0.07 | 1.39E−05 |
| rs4766805 | 12 | 117262969 | T | C | −0.09 | 2.12E−05 |
| rs10846635 | 12 | 124728014 | G | A | 0.04 | 8.81E−05 |
| rs10846742 | 12 | 125308682 | G | A | 0.09 | 3.64E−10 |
| rs11832357 | 12 | 131490712 | C | T | −0.10 | 5.44E−05 |
| rs7329258 | 13 | 43944759 | G | T | −0.04 | 1.39E−05 |
| rs75093613 | 13 | 72966621 | C | T | −0.18 | 1.60E−05 |
| rs61958028 | 13 | 74696521 | C | T | 0.17 | 3.62E−05 |
| rs144948159 | 13 | 82383886 | C | G | 0.26 | 8.07E−05 |
| rs9590312 | 13 | 96322107 | T | G | 0.05 | 2.55E−06 |
| rs3093930 | 14 | 20824181 | T | C | −0.04 | 9.76E−05 |
| rs10400725 | 14 | 21583378 | A | T | 0.08 | 9.94E−07 |
| rs1885151 | 14 | 35816139 | G | A | −0.04 | 2.88E−05 |
| rs147030733 | 14 | 55942656 | C | T | −0.11 | 9.39E−05 |
| rs4899393 | 14 | 71739924 | C | T | −0.05 | 9.28E−05 |
| rs56112295 | 14 | 105877057 | C | T | −0.07 | 3.55E−06 |
| rs112840248 | 15 | 42950885 | C | T | 0.08 | 1.54E−10 |
| rs877347 | 15 | 47587937 | G | A | 0.04 | 1.78E−05 |
| rs35349206 | 15 | 48726179 | C | A | −0.08 | 4.23E−07 |
| rs143575333 | 15 | 57127184 | A | G | 0.27 | 6.87E−05 |
| rs8032572 | 15 | 58311280 | G | A | 0.05 | 1.95E−06 |
| rs2043082 | 15 | 58674308 | G | A | −0.05 | 1.67E−06 |
| rs2070895 | 15 | 58723939 | G | A | −0.05 | 8.15E−05 |
| rs7173826 | 15 | 67528374 | T | G | −0.05 | 1.10E−05 |
| rs11072542 | 15 | 75634599 | G | A | −0.05 | 9.08E−05 |
| rs4243085 | 15 | 79057723 | G | C | 0.07 | 4.78E−09 |
| rs2521501 | 15 | 91437388 | A | T | −0.05 | 4.27E−05 |
| rs12910029 | 15 | 101449453 | C | T | −0.59 | 4.44E−06 |
| rs215089 | 16 | 16062603 | C | A | 0.05 | 3.07E−05 |
| rs1925500 | 16 | 20060063 | G | T | −0.04 | 3.91E−05 |
| rs56094641 | 16 | 53806453 | A | G | −0.07 | 5.77E−12 |
| rs8063219 | 16 | 69832912 | T | G | 0.05 | 4.88E−06 |
| rs9940520 | 16 | 73074012 | G | C | −0.06 | 2.38E−05 |
| rs11640724 | 16 | 75464086 | C | T | −0.04 | 5.27E−05 |
| rs4968211 | 17 | 7459062 | A | G | 0.10 | 3.49E−06 |
| rs12937280 | 17 | 14385160 | C | T | −0.15 | 2.32E−05 |
| rs570176374 | 17 | 30165552 | A | G | −0.12 | 4.43E−05 |
| rs8176216 | 17 | 41225781 | A | T | −0.06 | 1.66E−07 |
| rs58089049 | 17 | 43845480 | T | C | 0.05 | 1.57E−05 |
| rs8073039 | 17 | 57521208 | C | A | 0.04 | 7.64E−05 |
| rs9901606 | 17 | 57811407 | A | C | −0.05 | 7.00E−05 |
| rs8178824 | 17 | 64224775 | C | T | −0.13 | 1.10E−05 |
| rs7225126 | 17 | 68859060 | C | A | −0.04 | 3.16E−05 |
| rs7502390 | 17 | 79397611 | T | G | 0.06 | 5.41E−07 |
| rs607660 | 18 | 20129674 | C | T | 0.04 | 7.84E−05 |
| rs12453985 | 18 | 25713304 | C | T | 0.06 | 9.36E−07 |
| rs57214592 | 18 | 43949538 | C | T | 0.05 | 9.14E−05 |
| rs1942985 | 18 | 59576291 | A | G | 0.05 | 4.91E−05 |
| rs17070653 | 18 | 60775839 | C | T | −0.06 | 2.12E−05 |
| rs1827911 | 18 | 61403546 | G | A | 0.13 | 5.02E−05 |
| rs1421550 | 18 | 62021202 | C | T | −0.13 | 5.71E−05 |
| rs8113356 | 19 | 795816 | G | A | −0.06 | 2.53E−05 |
| rs7187 | 19 | 11275258 | A | G | 0.06 | 4.88E−08 |
| rs117855970 | 19 | 14990783 | A | T | −0.19 | 7.76E−05 |
| rs8101619 | 19 | 16410530 | C | T | −0.04 | 3.69E−05 |
| rs11083616 | 19 | 41865643 | G | A | −0.04 | 6.41E−05 |
| rs56012842 | 19 | 44036183 | G | T | 0.05 | 9.68E−05 |
| rs769449 | 19 | 45410002 | G | A | −0.08 | 6.91E−06 |
| rs553611 | 19 | 56703836 | G | T | 0.05 | 5.06E−05 |
| rs6108046 | 20 | 7704428 | C | T | −0.07 | 1.10E−05 |
| rs6040470 | 20 | 11229511 | G | T | 0.04 | 6.05E−05 |
| rs57671803 | 20 | 25258044 | A | C | 0.10 | 1.86E−05 |
| rs6060017 | 20 | 33313042 | C | A | −0.06 | 1.19E−05 |
| rs77575294 | 20 | 41332662 | A | G | 0.18 | 8.88E−05 |
| rs16983191 | 20 | 58390160 | G | C | −0.15 | 8.21E−05 |
| rs6062618 | 20 | 62682529 | T | G | 0.05 | 8.95E−05 |
| rs200589968 | 21 | 18925952 | C | T | 0.06 | 6.16E−05 |
| rs76542001 | 21 | 30544091 | G | A | 0.12 | 3.78E−06 |
| rs9980618 | 21 | 35600505 | C | T | 0.06 | 5.50E−05 |
| rs2410067 | 21 | 40295228 | T | C | −0.11 | 9.61E−06 |
| rs191153857 | 22 | 22796106 | G | A | −0.24 | 5.76E−05 |
| rs7287943 | 22 | 30892555 | C | A | 0.05 | 3.26E−05 |
| rs76548786 | 22 | 32615811 | T | G | −0.18 | 7.37E−05 |
| rs71313136 | 22 | 33038283 | T | A | 0.23 | 2.25E−07 |
| rs742152 | 22 | 37896749 | T | C | 0.06 | 9.76E−08 |
| rs4821688 | 22 | 38105810 | C | T | −0.07 | 4.01E−10 |
| rs11556482 | 22 | 45723807 | G | C | 0.05 | 2.04E−05 |

*Note*: SNP, single nucleotide polymorphism; Chr, chromosome; ALT, alternative allele; REF, reference allele; Beta, effect size estimate from GWAS summary statistics.
