# Supplementary figures and images for: Benefits of Better Cardiovascular Health for Calcific Aortic Valve Stenosis Stratified by Polygenic Risk Score
Source: Genomics Proteomics Bioinformatics. 2025 Nov 6;23(5):qzaf099. doi: 10.1093/gpbjnl/qzaf099 (PMC12812169; doi:10.1093/gpbjnl/qzaf099)

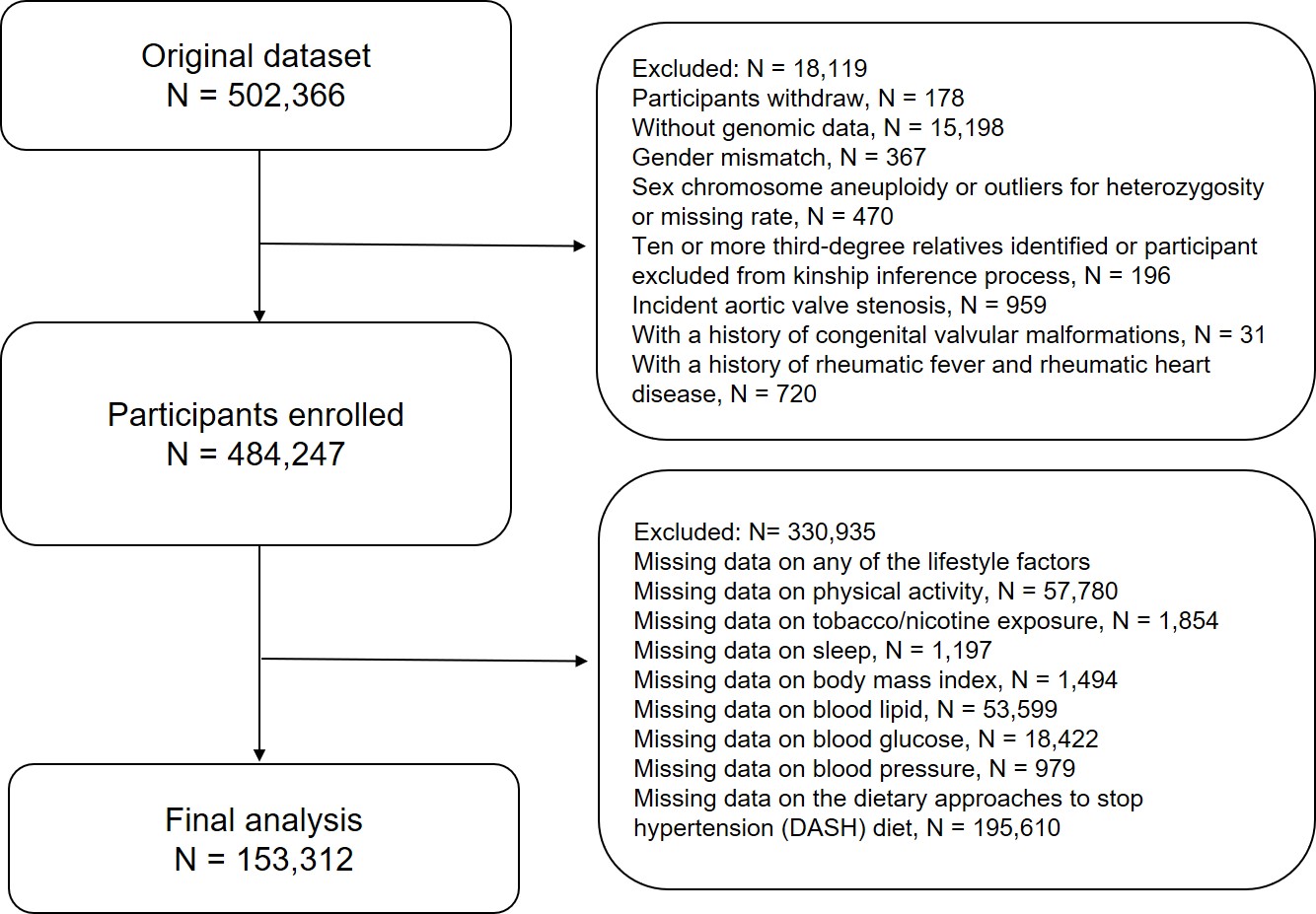

Supplement: qzaf099_Supplementary_Data [file qzaf099_supplementary_data.zip › Figure S1.jpg]

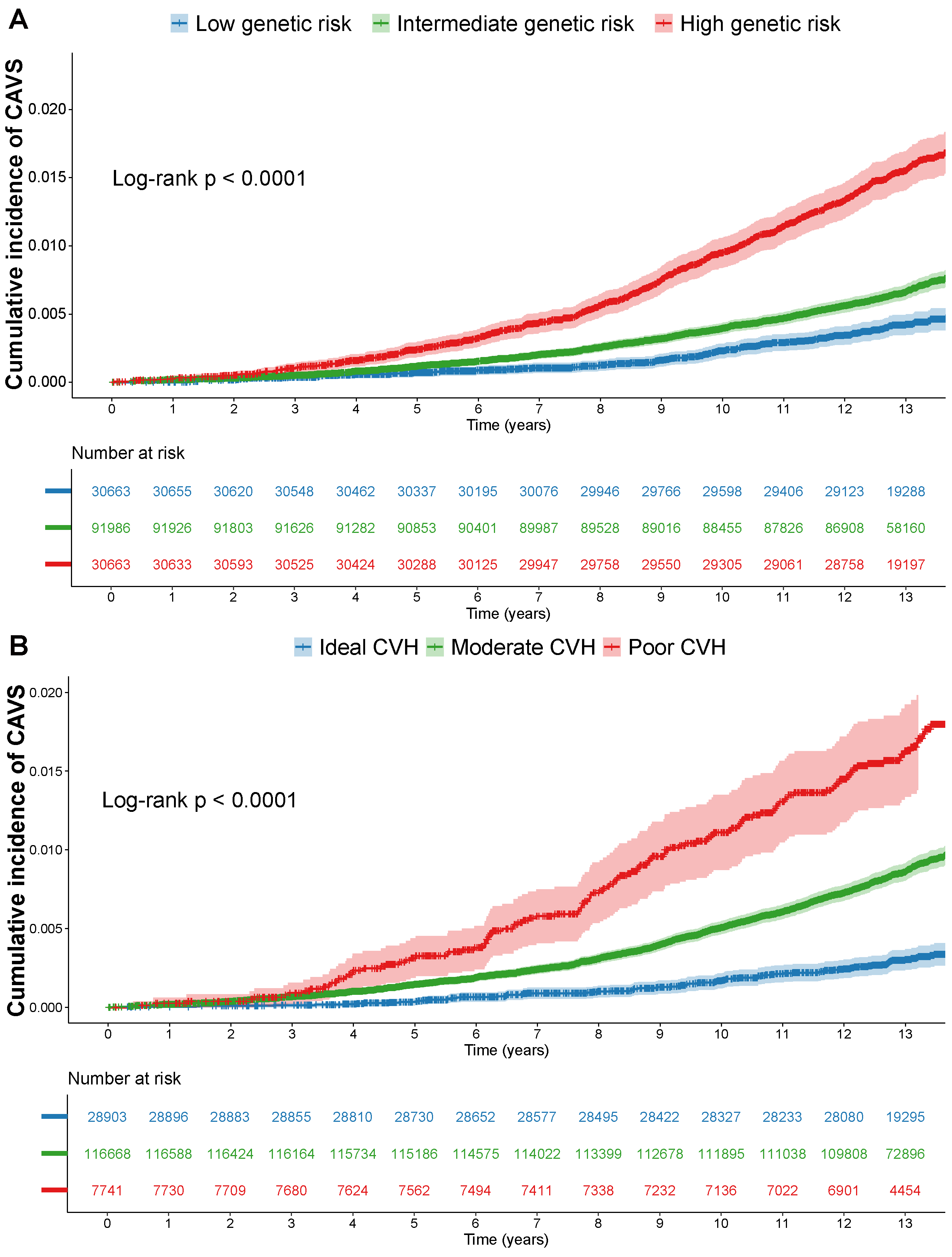

Supplement: qzaf099_Supplementary_Data [file qzaf099_supplementary_data.zip › Figure S3.tif]

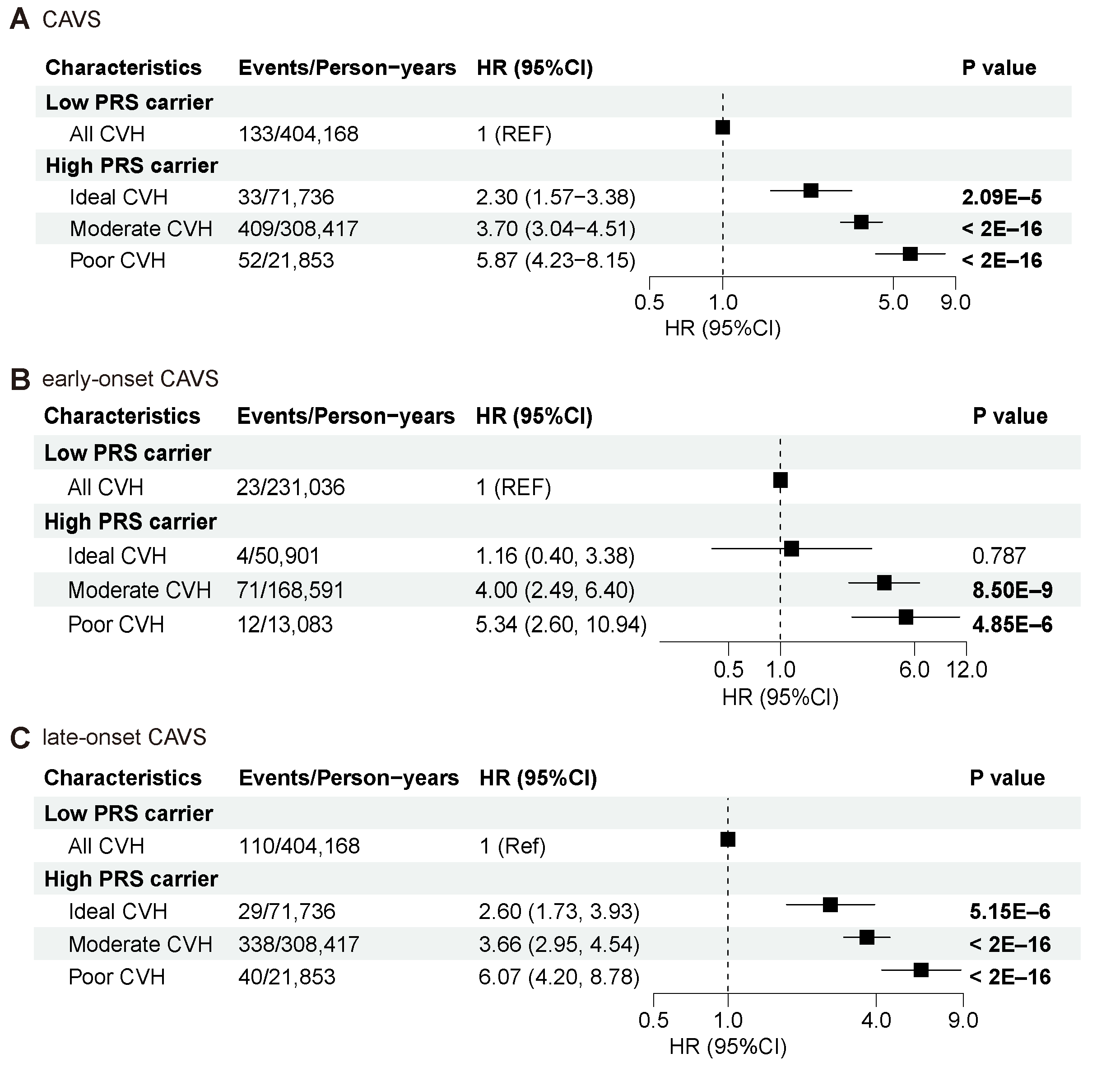

Supplement: qzaf099_Supplementary_Data [file qzaf099_supplementary_data.zip › Figure S4.tif]

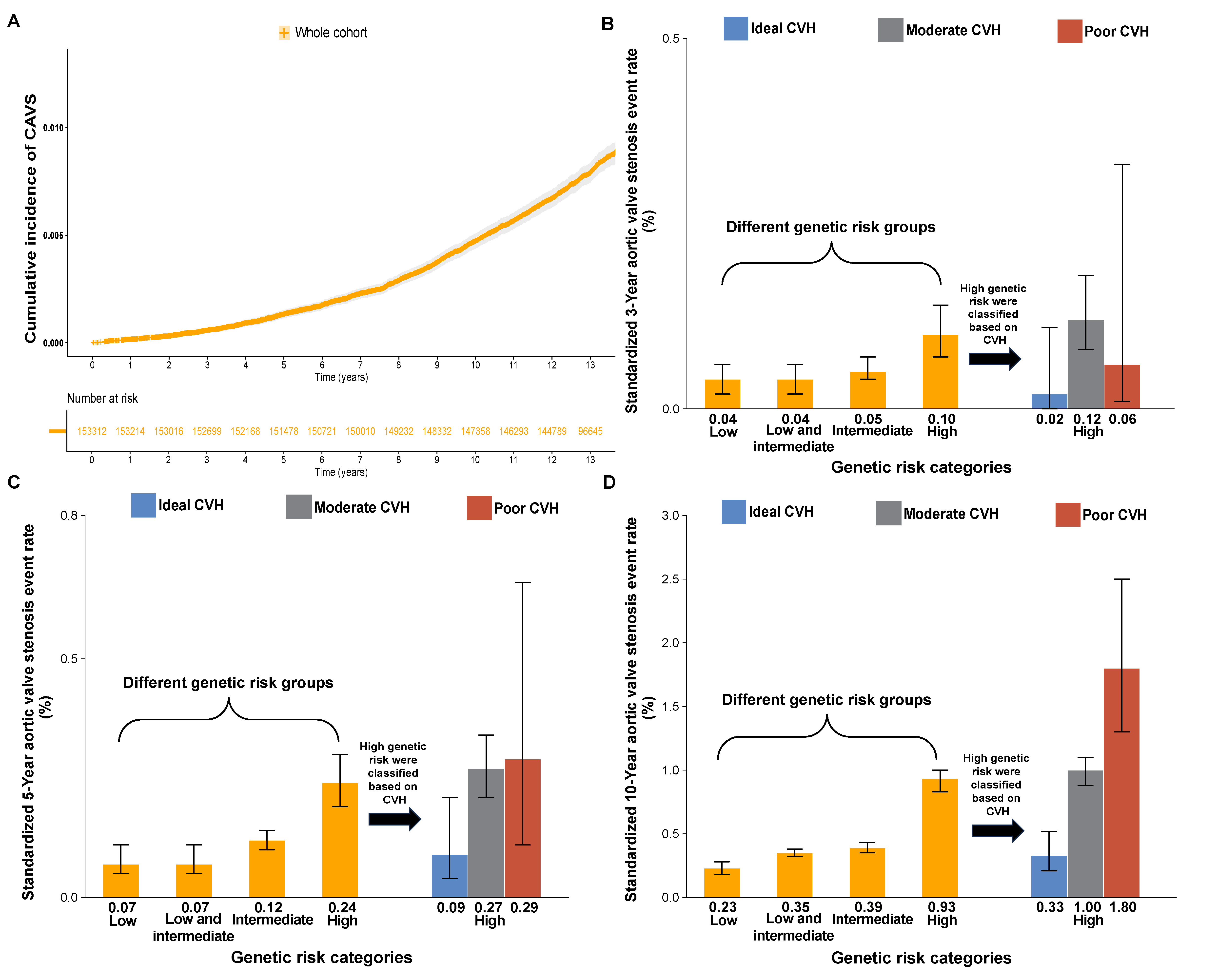

Supplement: qzaf099_Supplementary_Data [file qzaf099_supplementary_data.zip › Figure S6.tif]

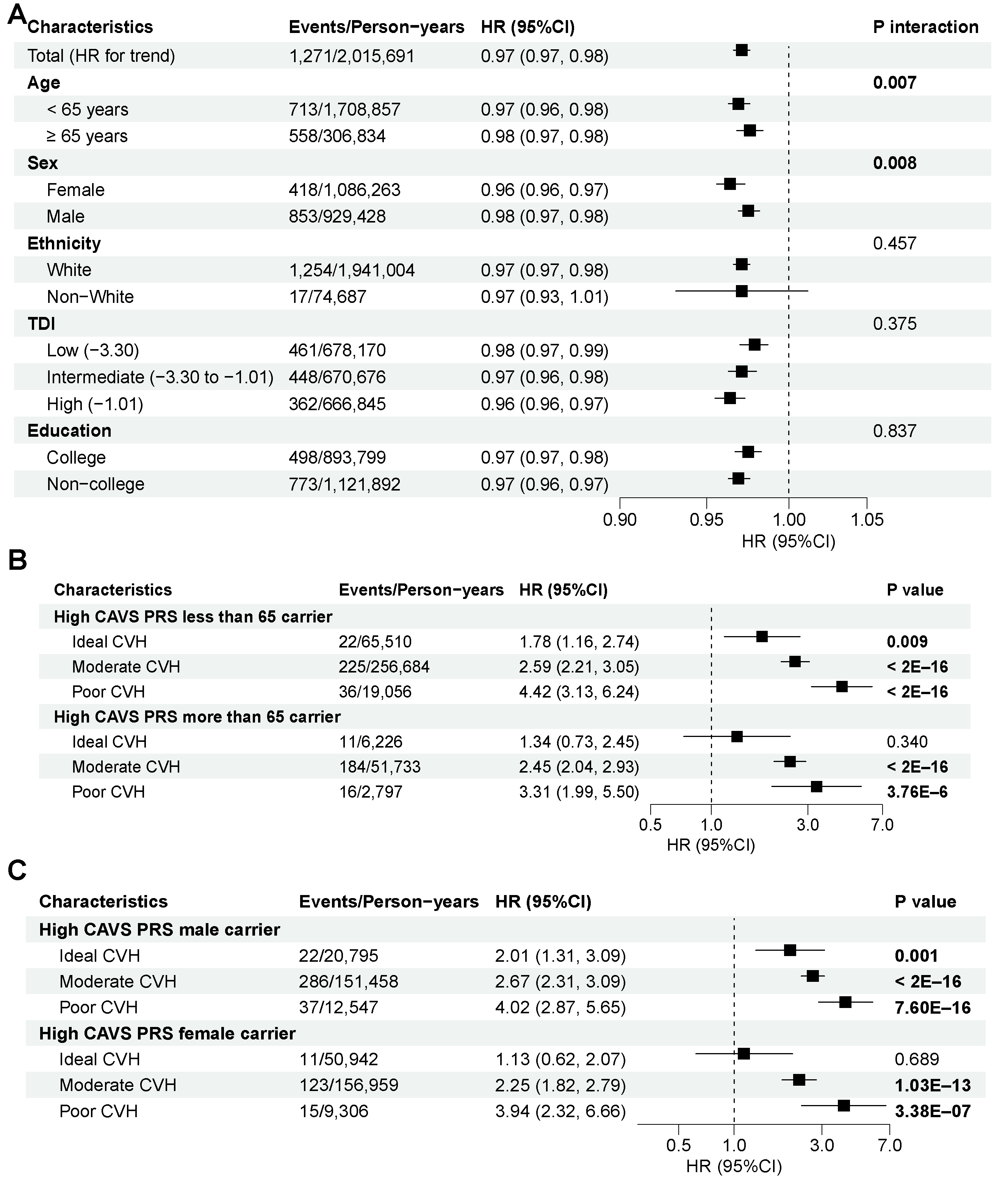

Supplement: qzaf099_Supplementary_Data [file qzaf099_supplementary_data.zip › Figure S7.tif]

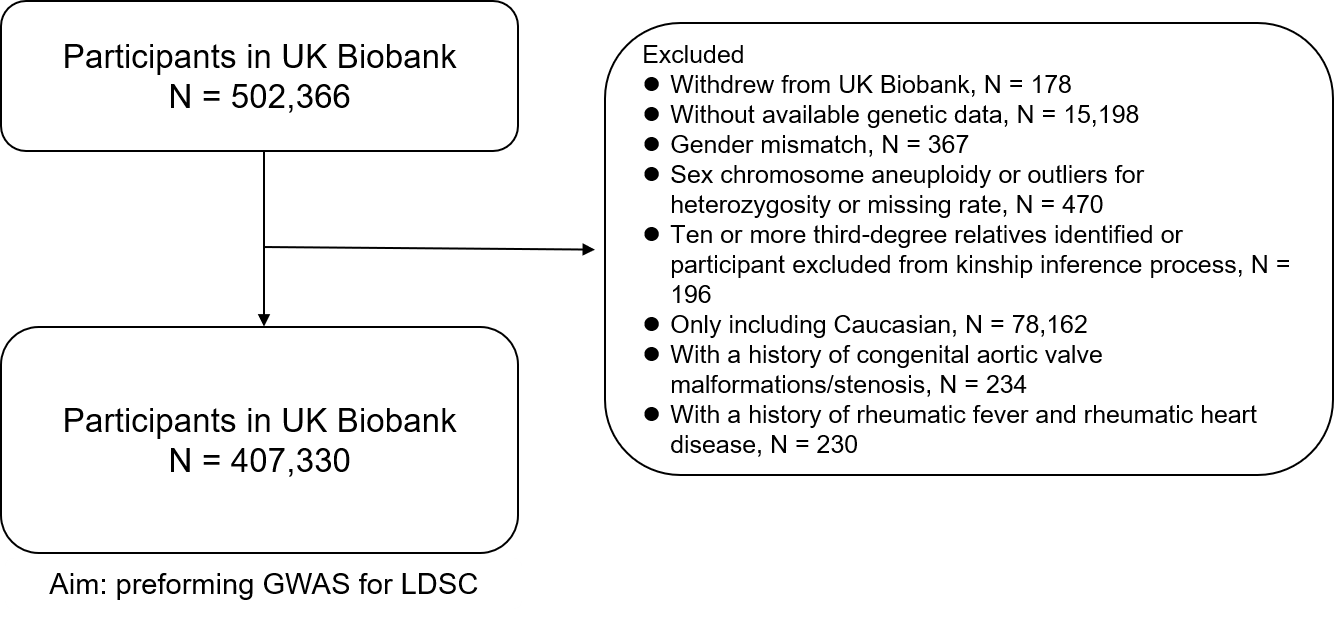

Supplement: qzaf099_Supplementary_Data [file qzaf099_supplementary_data.zip › Figure S8.tif]

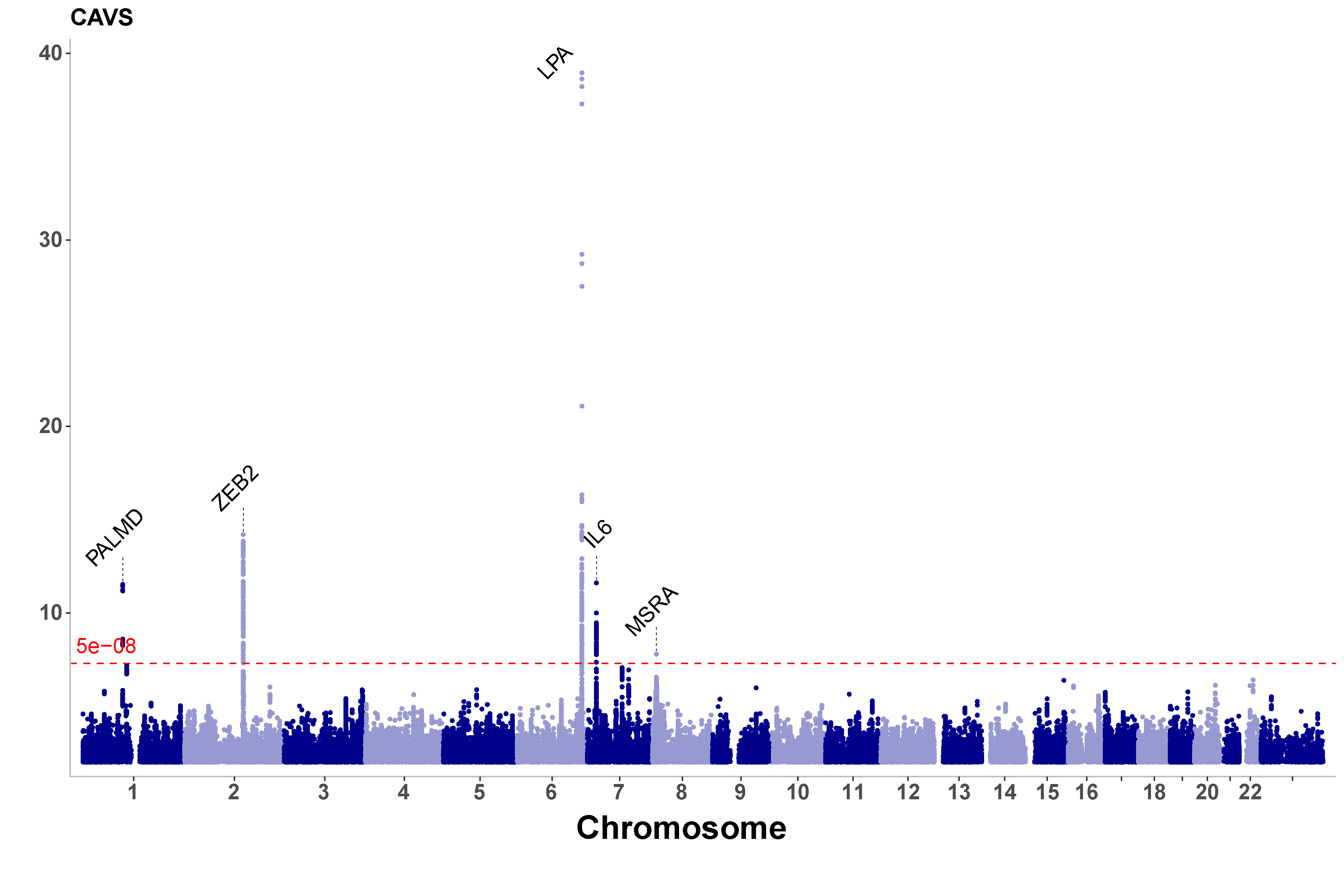

Supplement: qzaf099_Supplementary_Data [file qzaf099_supplementary_data.zip › Figure S9.tif]
